# Supplementary material for: Phosphatidic acid-dependent recruitment of microtubule motors to spherical supported lipid bilayers for in vitro motility assays
Source: Cell Rep. 2024 May 20;43(6):114252. doi: 10.1016/j.celrep.2024.114252 (PMC11220796; doi:10.1016/j.celrep.2024.114252)
Supplement: Document S1. Figures S1 and S2 and Table S1 [file mmc1.pdf]

Cell Reports, Volume *43*

Supplemental information

Phosphatidic acid-dependent recruitment of microtubule motors to spherical supported lipid bilayers for *in vitro* motility assays

Pankaj Kumar, Dwiteeya Chaudhury, Paulomi Sanghavi, Apurwa Meghna, and Roop Mallik

SUPPLEMENTARY INFORMATION

Supplementary Figures 1 and 2
Supplementary Table 1

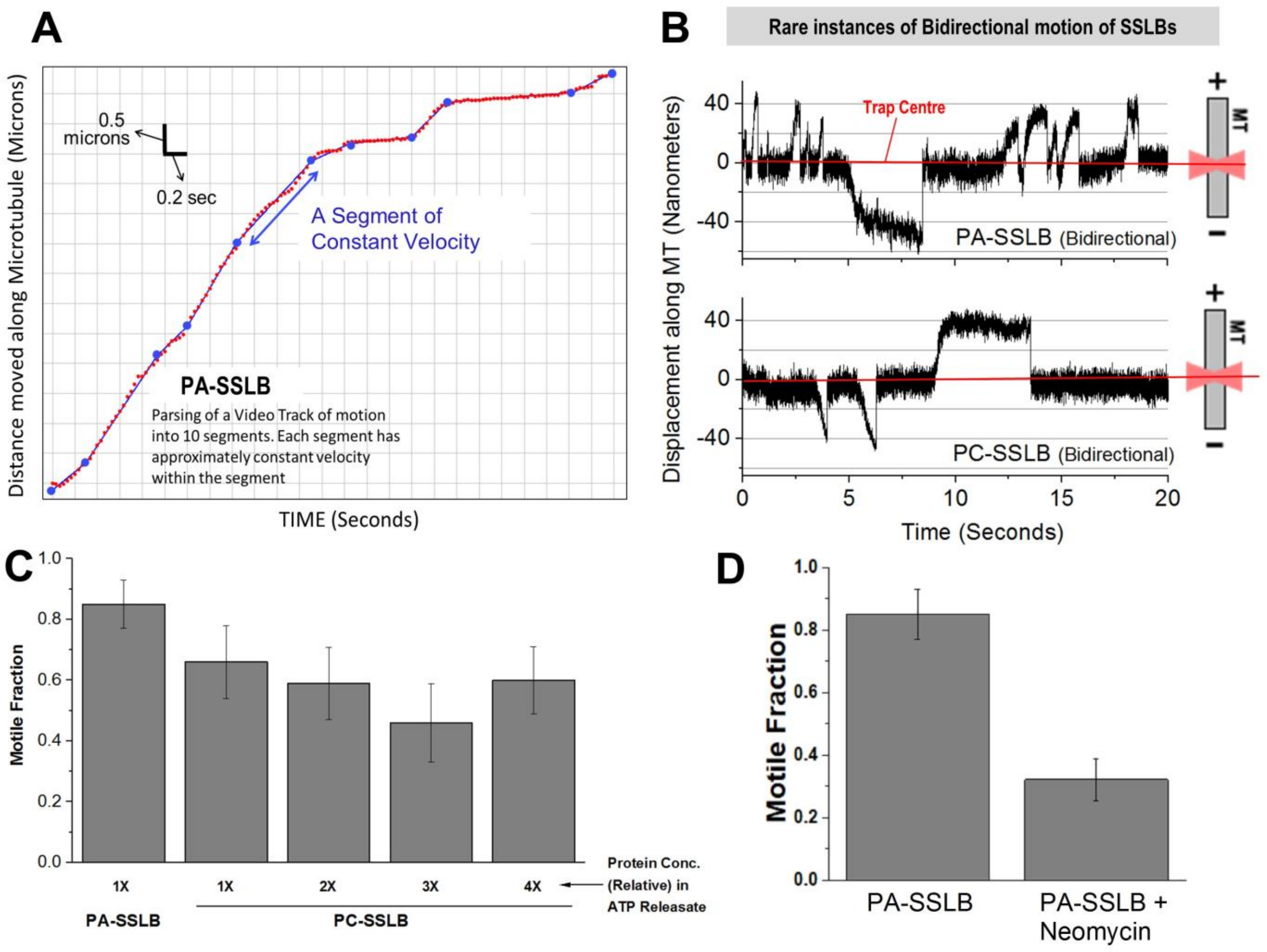

Supplementary Figure 1
Motion of SSLBs under different conditions

A. Distance versus time track (red dots) of a PA-SSLB moving freely along a MT (trap switched OFF). A Bayesian algorithm is applied to parse this track into 10 segments, each having an approximately constant velocity within the segment. The parsed segments are shown as large blue dots connected by a blue line. One such segment having a velocity (= slope) of 2 microns/sec is shown. The velocities obtained from such parsing are used in Figs 1B and 2B.

B. Bidirectional displacements against an optical trap for a PA-SSLB (top) and a PC-SSLB (bottom) incubated with *Dictyostelium* ATP releasate. These are the rare incidents where a given SSLB showed excursions in both directions against the trap. Red line represents the trap center.

C. Motile fraction of PA-SSLBs in presence of 1X (= 5µg total protein) amount of *Dictyostelium* ATP releasate. PC-SSLBs are incubated with increasing amounts of ATP releasate (1X to 4X; 5µg to 20µg protein). Error = $\sqrt{(P*(1-P)/N)}$. *P* is the motile fraction and *N* the number of SSLBs tested.

D. Motile fraction of PA-SSLBs in presence and absence of Neomycin (1mM). Error = $\sqrt{(P*(1-P)/N)}$. *P* is the motile fraction and *N* the number of SSLBs tested.

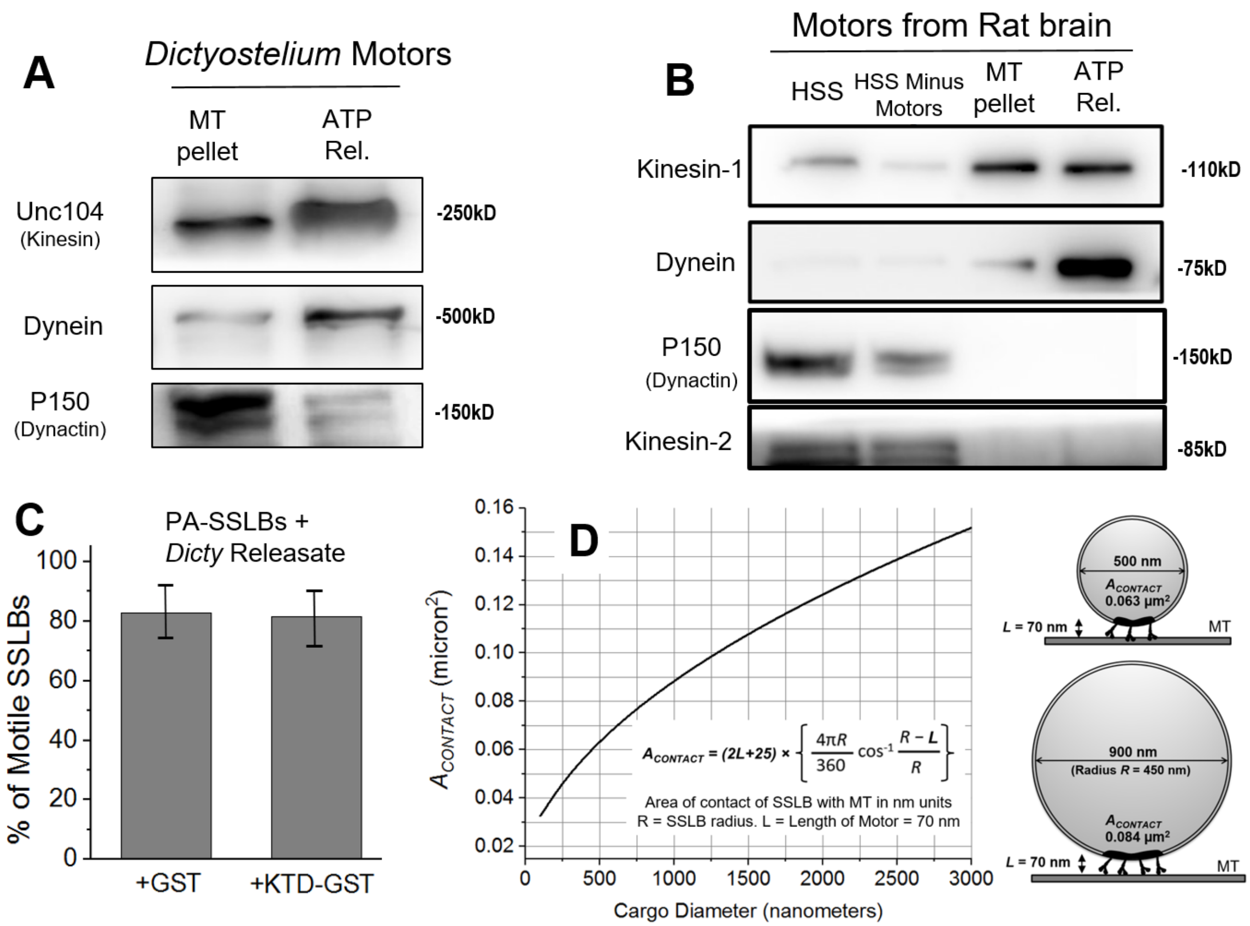

Supplementary Figure 2

Dynein, Dynactin, Kinesins probed in biochemical fractions, effect of KTD on *Dictyostelium* Motors, and Contact area of Motors on SSLBs.

A. Western blots probed for Dynein, Unc104, and Dynactin (P150) in the MT pellet and ATP releasate fractions from *Dictyostelium*. **MT pellet** is the pellet containing MTs after the ATP-release step has been done (see next). **ATP Rel.** is the supernatant after MTs have been pelleted down in the presence of 10mM ATP. ATP releasate is the motor-enriched fraction that is added to SSLBs in the motility assay. The MT pellet and ATP releasate lanes contain 75µg total protein per lane.

B. Western blots probed for Kinesin-1, Dynein, Dynactin (P150), and Kinesin-2 in different fractions obtained during the preparation of ATP releasate from rat brain. **HSS** High-speed supernatant (= clarified cytosol) after pelleting the cell membranes. *In-vitro* polymerized MTs are added to this HSS for binding motors and MAPs. **HSS Minus Motors** is the supernatant obtained after MT-bound motors and MAPs are pelleted down from the HSS. Equal volumes of HSS and HSS-Motors were loaded. **MT pellet** is the pellet containing MTs after the ATP-release step has been done (see next). **ATP Rel.** is the supernatant after MTs have been pelleted down in the presence of 10mM ATP. The MT pellet and ATP releasate lanes contain 15µg total protein per lane.

C. Motile fraction of PA-SSLBs prepared from *Dictyostelium* releasate in presence of control (GST) and Kinesin Tail Domain (KTD-GST). Error = $\sqrt{(P*(1-P)/N)}$. *P* is the motile fraction and *N* the number of SSLBs tested.

D. Graph represents change in $A_{CONTACT}$ with diameter of a spherical cargo (e.g. SSLB). Cartoon insets are schematics representing $A_{CONTACT}$, which is the region on a spherical cargo from where a motor of given size (L =70 nm) can access the underlying MT. The expression for $A_{CONTACT}$ is shown. See *Sanghavi et al Current Biology 2018* for more details. The two cargoes of different size and their attached motors are drawn to relative scale. Motors outside $A_{CONTACT}$ are not shown for the sake of clarity.

Supplementary Table 1

Selected Proteins detected by LC-MS/MS		Number of Unique Peptides detected		
**Accession Number**	**Description**	**ATP Releasate**	**PA-SSLBs (Sample 1)**	**PA-SSLBs (Sample 2)**
P05095	Alpha-actinin A	32	31	31
P54703	Cytoplasmic dynein 1 intermediate chain	3	8	11
Q54CI8	Cytoplasmic dynein 1 light intermediate chain 1	6	7	9
Q54VD6	Dynactin 150 kDa subunit	7	30	29
Q54N50	Dynactin subunit 2	7	13	16
Q55F94	Dynamin-like protein A	16	26	27
Q54MH8	Dynamin-like protein B	8	13	16
Q54VS0	Dynactin subunit 4	3	3	4
P34036	Dynein heavy chain, cytoplasmic	77	153	174
Q9NGQ2	Kinesin-related protein 1	57	79	97
Q6S001	Kinesin-related protein 11	6	8	12
Q54UC9	Kinesin-related protein 3	28	38	34
Q8T135	Kinesin-related protein 5	11	15	17
Q6S003	Kinesin-related protein 8	9	19	25
P32255	Tubulin alpha chain	7	15	13
P32256	Tubulin beta chain	11	16	18

Supplementary Table 1.

LC-MS/MS proteomics of ATP releasate from *Dictyostelium* and PA-SSLBs incubated with ATP releasate.

Selected proteins that were detected using mass spectrometry (LC-MS/MS) in ATP releasate from *Dictyostelium* and on PA-SSLBs incubated with ATP releasate from *Dictyostelium.* PA-SSLBs were prepared, tested and analysed in duplicate (Sample 1 and Sample 2). A complete list of detected proteins is available from the PRIDE database with the dataset identifier PXD051777.
