# Supplementary material for: Phosphatidic acid-dependent recruitment of microtubule motors to spherical supported lipid bilayers for in vitro motility assays
Source: Cell Rep. 2024 May 20;43(6):114252. doi: 10.1016/j.celrep.2024.114252 (PMC11220796; doi:10.1016/j.celrep.2024.114252)
Supplement: Document S2. Article plus supplemental information [file mmc6.pdf]

Cell Reports

Article

Phosphatidic acid-dependent recruitment of microtubule motors to spherical supported lipid bilayers for *in vitro* motility assays

Graphical abstract

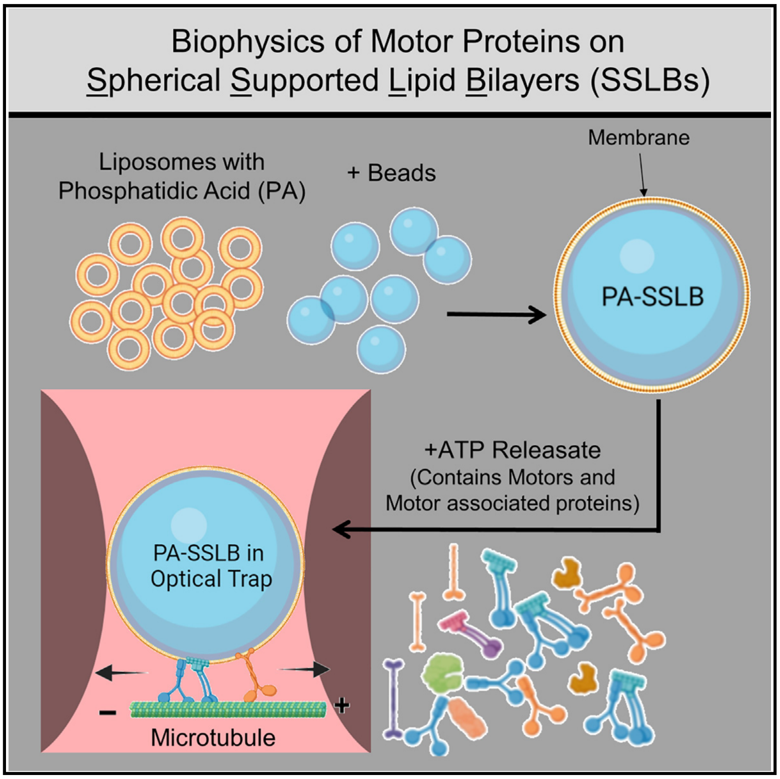

Highlights

- Beads enclosed in lipid membrane containing phosphatidic acid as "cargo" for motor proteins
- Cargoes move robustly on microtubules driven by dynein or kinesin-1 motors *in vitro*
- Larger cargoes reverse more, suggesting how traffic jams may be avoided inside cells
- "Locking" or "non-locking" motor inhibitors to resolve a paradox in intracellular transport

Authors

Pankaj Kumar, Dwiteeya Chaudhury, Paulomi Sanghavi, Apurwa Meghna, Roop Mallik

Correspondence

rmallik@iitb.ac.in

In brief

Kumar et al. prepare beads enclosed within a membrane containing phosphatidic acid as a "cargo" for motor proteins. Phosphatidic acid recruits dynein or kinesin-1 to the cargo, depending on the source of motors. Larger cargoes reverse more, and inhibitors may "lock" a motor to block overall motion of the cargo.

Kumar et al., 2024, Cell Reports *43*, 114252
June 25, 2024 © 2024 The Author(s). Published by Elsevier Inc.
https://doi.org/10.1016/j.celrep.2024.114252

CellPress

Cell Reports

CellPress OPEN ACCESS

Article

Phosphatidic acid-dependent recruitment of microtubule motors to spherical supported lipid bilayers for *in vitro* motility assays

Pankaj Kumar,[1] Dwiteeya Chaudhury,[2] Paulomi Sanghavi,[2] Apurwa Meghna,[2] and Roop Mallik[2,3,*]
[1]Department of Biological Sciences, Tata Institute of Fundamental Research, Mumbai 400005, India
[2]Department of Biosciences and Bioengineering, Indian Institute of Technology Bombay, Mumbai 400076, India
[3]Lead contact
*Correspondence: rmallik@iitb.ac.in
https://doi.org/10.1016/j.celrep.2024.114252

SUMMARY

Motor proteins transport diverse membrane-bound vesicles along microtubules inside cells. How specific lipids, particularly rare lipids, on the membrane recruit and activate motors is poorly understood. To address this, we prepare spherical supported lipid bilayers (SSLBs) consisting of a latex bead enclosed within a membrane of desired lipid composition. SSLBs containing phosphatidic acid recruit dynein when incubated with *Dictyostelium* fractions but kinesin-1 when incubated with rat brain fractions. These SSLBs allow controlled biophysical investigation of membrane-bound motors along with their regulators at the single-cargo level *in vitro*. Optical trapping of single SSLBs reveals that motor-specific inhibitors can "lock" a motor to a microtubule, explaining the paradoxical arrest of overall cargo transport by such inhibitors. Increasing their size causes SSLBs to reverse direction more frequently, relevant to how large cargoes may navigate inside cells. These studies are relevant to understand how unidirectional or bidirectional motion of vesicles might be generated.

INTRODUCTION

Intracellular transport of vesicular cargoes (e.g., endosomes, mitochondria, and phagosomes) requires force from motor proteins that can attach to the cargo membrane directly or via adaptor proteins.[1,2] Biophysical function of motors has been investigated *in vitro* using microtubule-gliding and bead-motility assays.[3] In both assays, however, the motor is usually attached *sans* membrane to a non-physiological surface such as a glass cover slip or a plastic bead. How membrane lipids affect motor recruitment and activity (i.e., force), and the implications thereof, are therefore poorly understood.[2] The lipid membrane is not just a passive surface, rather, it can recruit specific motors and modulate how a motor functions on the cargo. For example, the kinesin-3 family member KIF16B binds to phosphatidylinositol-3-phosphate via a PX motif, whereas the KIF1A/Unc104 motor binds to phosphatidylinositol-4,5-bisphosphate via its PH domain.[4] Microtubule-gliding assays suggest that cholesterol prevents slippage of kinesin against opposing force.[5] A collection of myosin-V motors is able to transport synthetic liposomes faster when the underlying lipid is in a fluid state, perhaps because trailing myosins detach more easily in this situation.[6] Similar mechanisms are also suggested for the kinesin-1 motor.[7] Transport inside cells can also be affected profoundly by the lipid composition of endosomes/phagosomes,[8,9] metalloprotease-containing vesicles,[10] and lipid droplets.[11] The importance of lipid-to-motor interactions and their potential implications to cell/physiological functions has been reviewed.[2]

In the context of this study, phosphatidic acid (PA) is a rare but highly bioactive phospholipid with established functions in cell signaling,[12] vesicular trafficking,[10,11,13,14] and physiology/lipid homeostasis.[11,15] PA is an acidic phospholipid with a small negatively charged head group and a conical shape. Thus, PA interacts with a variety of proteins (including motors) through a unique combination of electrostatic, hydrogen-bonding, and curvature-dependent mechanisms.[12,14] In metastatic tumor cells, the conventional kinesin (kinesin-1) motor binds directly to PA on the membrane of metalloprotease-containing vesicles. Kinesin-1 transports these vesicles to the cell periphery, following which metalloproteases are secreted out to invade neighboring tissue.[10] We have shown that PA-dependent recruitment of kinesin-1 to lipid droplets in the liver is important for lipoprotein secretion and systemic lipid homeostasis.[11,16] Taken together, PA connects vesicle transport to tumor proliferation, systemic lipid homeostasis, and possibly other unknown biological pathways. We emphasize that PA is a rare phospholipid that exists at a few mole-percent in membrane lipids but has high affinity to specific proteins.[12,14] Therefore, even small changes in PA can have profound effect on cell/physiological functions.[10,11] The above findings reveal novel disease-relevant roles for PA-motor interactions, but these are not easy to dissect using a completely *in vivo* approach. Such studies could be advanced if certain aspects of PA-motor interactions could be reconstituted outside cells.

With this motivation, here, we prepare artificial cargoes that consist of latex beads coated with a membrane containing

© 2024 The Author(s). Published by Elsevier Inc.
This is an open access article under the CC BY license (http://creativecommons.org/licenses/by/4.0/).

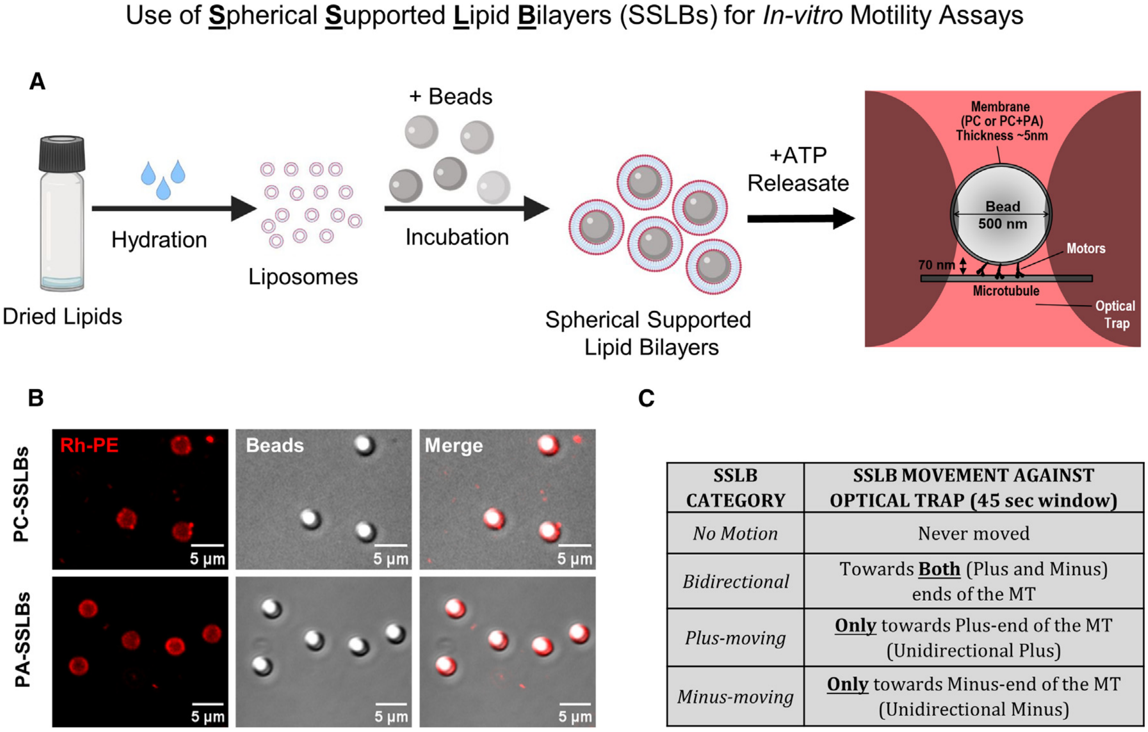

Figure 1. Preparation and *in vitro* motility of spherical supported lipid bilayers

(A) Schematic for preparation of spherical supported lipid bilayers (SSLBs) from liposomes. A lipid mix of desired composition (only PC or PC + 5 mol % PA) is dried into a lipid film and hydrated to obtain liposomes. Liposomes are then incubated with latex beads to form SSLBs. Buoyant SSLBs are separated by centrifugation and incubated with ATP releasate to recruit motors and other proteins onto the SSLB membrane. SSLBs are then introduced into a flow chamber having microtubules adhered to the cover slip surface for observing free SSLB motion or force generation by SSLB-bound motors against an optical trap (focused laser beam).

(B) Confocal image of 3-μm (diameter) PC-SSLBs and PA-SSLBs. The red signal (leftmost panel and merge) is rhodamine-PE, present in 0.01 mol % to visualize the lipid membrane.

(C) Categorization of *in vitro* SSLB motion depending on their direction of motion along microtubules.

phosphatidylcholine (PC) and PA. We choose PC, as it is an essential and abundant structural lipid that makes up >50% of phospholipids in most cellular membranes. These bead-containing membranous structures are called spherical supported lipid bilayers (SSLBs; Figure 1A). Others have studied dynein and kinesin function on SSLBs.[5,7,17] Recently, purified dynein-dynactin-BicD (DDB) and KIF16B kinesin were shown to transport unilamellar vesicles of ~130-nm diameter in a bidirectional manner.[17] Based on numerical simulations, these authors suggested that vesicles having around 8 DDBs and 14 kinesins exhibit intermittent pauses and reversals. These studies used purified motors that were attached to functionalized lipids via artificial linkages and may therefore have excluded motor-associated regulatory proteins that affect motor function.[1,18] Further, the artificial motor-lipid linkages may modify force generation by motors/motor-complexes.

To study how motors function on lipids in a more native-like context, we recruit motors to SSLBs simply by incubating the SSLBs with an ATP releasate that is enriched in motors and motor-associated proteins. By trapping individual SSLBs in an optical trap and holding them close to a microtubule (MT), we directly study how the incorporation of PA changes the activity (i.e., the force) from motors on the SSLB. These optical trapping results are combined with biochemical studies to infer how PA may recruit/activate different kinds of motors on a membrane. PA causes motor-driven SSLB motion of distinct varieties, depending on the source of the ATP releasate. PA-containing SSLBs (PA-SSLBs) recruit dynein and dynactin from *Dictyostelium* releasate to drive minus-directed SSLB motion. However, interestingly, PA-SSLBs recruit kinesin-1 from rat brain releasate to drive plus-directed SSLB motion. Irrespective of the source and type of motors, PA significantly enhances motion and force generation by motors. Our biophysical data suggest how specific inhibitors against one type of motor may block overall cargo transport inside cells. We find that larger SSLBs reverse their direction of motion more frequently, this being relevant to how large cargoes may navigate around inside cells. Lastly, SSLBs provide some insight into how unidirectional versus bidirectional transport might be generated for cellular cargoes.

RESULTS

Preparation of motor-bound SSLBs for optical trapping and biochemical assays

We first prepared liposomes of a defined lipid composition doped with trace amounts of the fluorescent lipid rhodamine-phosphatidylethanolamine (0.01% Rh-PE). These liposomes

CellPress
OPEN ACCESS

were then deposited onto a latex bead of 500nm diameter, as described in Figure 1A (also see STAR Methods). Imaging of Rh-PE showed the SSLB membrane as a ring-like structure enclosing the bead tightly in confocal sections (Figure 1B). SSLBs of different lipid composition (see below) were next incubated with ATP-releasate that was prepared from *Dictyostelium* or from rat brain (see STAR Methods). Briefly, we incubated clarified cytosol with MTs in the absence of ATP to allow binding of motors and MT-associated proteins to the MT. MTs were pelleted, after which ATP was added to release motors into the supernatant, thus yielding the ATP-releasate. SSLBs were then incubated with ATP-releasate on ice for 5 min to allow attachment of proteins (including motors) to lipids on the SSLB membrane (Figure 1A). This method avoids multiple rounds of centrifugation that is required for purification of endogenous motors from mammalian cells/tissues (e.g., see Bingham et al.[19]), during which enzymatic activity of motors may degrade.

As demonstrated by us and others earlier using latex bead phagosomes,[20–22] the buoyancy of latex beads also allows rapid separation of a large and highly pure fraction of SSLBs that can then be subjected to bulk biochemical assays to identify SSLB-associated proteins. Further, the spherical refractile beads enclosed within the thin (~5 nm) membrane makes the SSLB an ideal cargo for optical trap based force measurements.[7,21] SSLB motion was assayed by flowing in the SSLBs along with the ATP-releasate onto *in vitro*-polymerized MTs that had been adhered previously to a cover slip inside a flow cell. Individual SSLBs were caught in the trap and held close to an MT. The underlying MT and SSLB were visualized in a differential interference contrast microscope equipped with an optical trap as described earlier.[23] We observed force generation by SSLB-bound motors against the trap, as well as free motion of SSLBs after switching off the trap. We used MTs with a biotinylated minus end marked by streptavidin-coated beads (STAR Methods) to identify the MT polarity.[24] Using polarity-labeled MTs allowed us to determine the activity of kinesin versus dynein in real time on SSLBs and thus to separate the SSLBs into four possible categories based on their motion (Figure 1C; also see next section).

PA enhances dynein-driven SSLB motion with ATP releasate from *Dictyostelium*

To probe the effect of PA, SSLBs were prepared using only PC (called PC-SSLBs) or with 95 mol % of PC + 5 mol % of PA (PA-SSLBs). PA levels in cell membranes are between 2 and 4.4 mol %,[25] and previous studies have used PC:PA in similar ratio.[10,11] SSLBs incubated with *Dictyostelium* ATP releasate were held above an MT with the trap to engineer motility events. Figure 2A shows representative displacement versus time tracks of the free motion of PC and PA-SSLBs along MTs. The optical trap was switched off right after an SSLB started to move in this experiment. Free motion of representative PC-SSLBs and PA-SSLBs is also shown in Videos S1 and S2. The video tracks were parsed into segments of constant velocity,[26,27] as shown for a representative track in Figure S1A. Based on such parsing, PA-SSLBs usually moved toward the MT minus end with higher velocities compared to PC-SSLBs (Figure 2B). Overall, PA-SSLBs exhibited qualitatively improved motion that was longer, smoother, and faster.

We next assayed force generation by motors on individual SSLBs against the trap on polarity-labeled MTs. These events were recorded using a quadrant photo detector[23] at high spatial and temporal resolution (~5 nm; 1-kHz sampling frequency), making it unlikely that any transient activity of motors would be missed. Studying SSLBs in an optical trap on polarity-labeled MTs allows rapid, real-time measurement of the force (i.e., function) of lipid-bound kinesin or dynein motors. Figure 2C shows 20 s of optical trap data for a "plus-directed" PC-SSLB and a "minus-directed" PA-SSLB, so called because each of these SSLBs showed repeated stalls against the trap in only one direction. Examples of "bidirectional" motion are shown in Figure S1B. Most PA-SSLBs exhibited repeated minus-directed stalls in rapid succession (Figure 2C, lower panel). PA-SSLBs also moved farther away from the trap center compared to PC-SSLBs, suggesting higher force from motors on PA-SSLBs. Figure 2D shows the distribution of SSLBs as determined from optical trapping data based on the SSLB categorization described in Figure 1C. Note that 34% of PC-SSLBs were non-motile, but this fraction was only 9% for PA-SSLBs. Thus, a high overall fraction (~91%) of SSLBs showed robust activity of motors in presence of PA. Even after incubation of PC-SSLBs with ATP releasate containing 4-fold more proteins (and therefore 4-fold more motors), the motile fraction of PC-SSLBs remained lower than PA-SSLBs (Figure S1C). Taken together, PA significantly improved the activity of *Dictyostelium* motors on SSLBs as measured by different parameters. This improvement could stem from enhanced recruitment of motors by PA and/or enhanced activity of the motors that are bound to PA.

Unc104, a processive kinesin-3 family motor is the major plus-directed motor for long-range transport in *Dictyostelium.*[4] A significant fraction of plus-directed motion for PC-SSLBs is likely driven by Unc104. PA-SSLBs, which exhibit improved overall motility, also showed a remarkable reversal in the direction of motion compared to PC-SSLBs. The majority of PA-SSLBs (64%) were minus directed, with only 15% being plus directed and 12% bidirectional (Figure 2D). Thus, a minus-directed motor is highly activated by addition of PA to SSLBs. This motor is likely dynein because processive minus-directed kinesins are not found in the genome of *Dictyostelium.*[28] In an earlier report, we have found that dynein motors cluster into cholesterol-rich microdomains on late phagosomes, yielding robust minus-directed motion and frequent stalls.[9] The PA-SSLB motion seen here therefore has some qualitative similarities to late phagosome motion, although PA-SSLBs do not contain cholesterol.

PA recruits the *Dictyostelium* dynein-dynactin complex to SSLBs

Dictyostelium discoideum ATP releasate contained Unc104, dynein, and dynactin (Figure S2A). To evaluate the affinity of these motors to lipids on the SSLB, we conducted an "SSLB pull-down" experiment. PC or PA-SSLBs were incubated with ATP-releasate from *Dictyostelium*. The SSLBs were then separated by centrifugation, washed, and probed by western blotting for lipid-bound motors. PA-SSLBs recruited significantly more dynein compared to PC-SSLBs, but Unc104 kinesin was recruited equally to PC and PA-SSLBs (Figures 2E and 2F). To

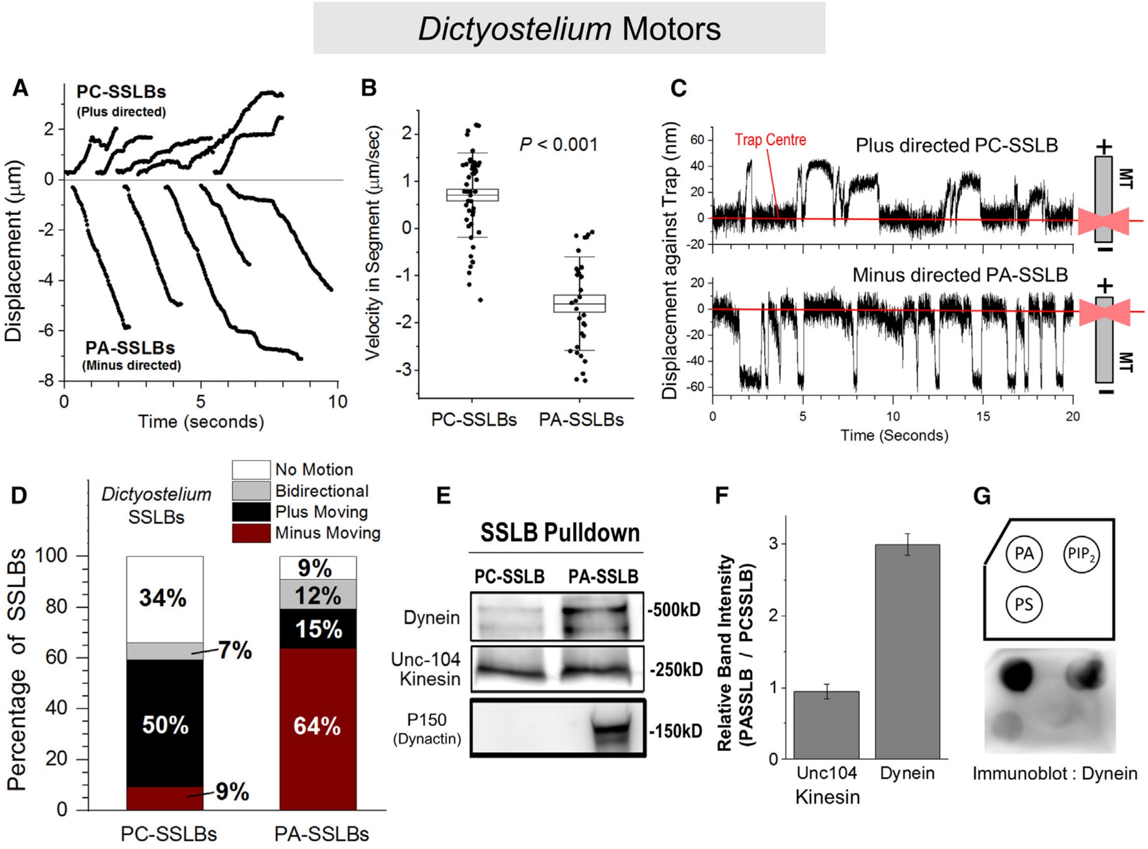

Figure 2. Phosphatidic acid enhances minus-directed motion of SSLBs prepared using *Dictyostelium* ATP releasate

(A) Representative tracks for free runs of PC-SSLBs and PA-SSLBs shown as a displacement versus time graph. Tracks have been offset by arbitrary amounts for ease of representation.

(B) Plot representing the velocity within constant-velocity segments for PC and PA-SSLBs (see main text). PA-SSLBs have higher (minus-directed) average velocity than PC-SSLBs. Error bars represent SD, and boxes represent SEM of the data. The *p* value is obtained using Student's t test by comparing the magnitude of the mean of segment velocities (direction of motion was ignored during comparison).

(C) Displacement vs. time graph for a PC-SSLB moving toward plus and PA-SSLB moving toward minus direction against an optical trap. Schematics on the right depict the experimental geometry and MT polarity. Red lines indicate the center of the trap (zero position). These direction-specific tracks are representative of the dominant direction of motion for PC-SSLBs (plus) and PA-SSLBs (minus). The PA-SSLB shows more frequent force generation events compared to the PC-SSLB. The PA-SSLB also moves out farther (~60 nm), and therefore, motors on the PA-SSLB generate higher force compared to the PC-SSLB (~40 nm). The trap stiffness is 0.07 ± 0.01 pN/nm.

(D) Categorization of SSLBs based on their motion on polarity-labeled MTs. Very few PA-SSLBs are immotile compared to PC-SSLBs. Note the drastic increase in minus-moving SSLBs upon addition of PA. A total of 32 PCs and 95 PA-SSLBs were analyzed.

(E) Western blots representing motors retained on SSLBs after an "SSLB pull-down." Number of PA-SSLBs and PC-SSLBs were normalized using the optical density at 500 nm (STAR Methods). PC and PA-SSLBs were incubated with *Dictyostelium* ATP releasate, washed, and probed for *Dictyostelium* dynein, *Dictyostelium* Unc104 kinesin, and *Dictyostelium* dynactin.

(F) Quantitation of motor binding to PC and PA-SSLB. PA has no significant effect on Unc104 recruitment to SSLBs. However, dynein amount on PA-SSLBs is ~3 times of that bound to PC-SSLBs (mean ± SEM, $N = 3$).

(G) Lipid blot using *Dictyostelium* ATP releasate spotted on phosphatidic acid (PA), phosphatidylinositol-4,5-bisphosphate (PIP_2), and phosphatidylserine (PS). Dynein is probed using an antibody.

further probe the mechanism of dynein binding, we subjected *Dictyostelium* ATP releasate and PA-SSLBs to liquid chromatography-coupled tandem mass spectrometry (LC-MS/MS). We detected a homolog of the human α-actinin protein (accession number P05095) that is believed to be a candidate for prototypic spectrin in *Dictyostelium.*[29] This protein has two calponin homology domains that are also present in spectrin. The number of peptides matched for this protein was 31 in duplicate samples of PA-SSLBs (Table S1).

Muresan et al.[30] found that spectrin binds to acidic phospholipids such as PA and also to dynactin via ARP1, thus recruiting dynein-dynactin to vesicles. They also showed that the drug neomycin blocks spectrin-to-PA binding, thus removing dynein-dynactin from liposomes and reducing dynein-driven motion. Indeed, adding neomycin to the motility mixture drastically reduced PA-SSLB motion (Figure S1D), suggesting that a spectrin-like protein recruits dynein-dynactin to PA on SSLBs. These findings are consistent with the detection of dynactin

CellPress
OPEN ACCESS

by western blotting on PA-SSLBs but not on PC-SSLBs (Figure 2E). The interaction between dynein and dynactin, and therefore the motion driven by dynein-dynactin complexes, can also be disrupted by a peptide that mimics the dynein intermediate chain.[31,32] We have cloned the dynein intermediate chain from *Dictyostelium* and have shown that this peptide specifically inhibits minus-directed motion driven by motors from *Dictyostelium* ATP releasate.[21] Taken together, minus-directed PA-SSLB motion appears driven by the dynein-dynactin complex that is bound to PA via a protein in *Dictyostelium* that has homology to spectrin. While 50% of PC-SSLBs were plus-directed, very few (only 15%) PA-SSLBs were plus-directed (Figure 2D). It therefore appears that the excess dynein-dynactin recruited to PA-SSLBs confers minus-directed motility to PA-SSLBs by dominating over kinesins. While this may be true for dynein activation through PA, other lipids could activate specific motors using different mechanisms. Note that we also detected several kinesin-related proteins in *Dictyostelium* ATP releasate and on PA-SSLBs by LC-MS/MS, including the Unc104 kinesin (Table S1).

To better understand lipid-motor affinities, we next performed lipid blot assays using *Dictyostelium* ATP releasate. PA is an acidic phospholipid with a negatively charged head group and a conical shape.[12,14] While other acidic phospholipids such as phosphatidylserine (PS) and phosphatidylinositol-4,5-bisphosphate (PIP_2) also bound to *Dictyostelium* dynein, their affinity was lower than PA (Figure 2G). It is therefore possible that in addition to its negative charge, the conical shape of PA has a role in dynein-dynactin binding. Taken together, these data suggest that PA-SSLBs can be used to recruit dynein-dynactin from *Dictyostelium* for analyzing dynein function on a lipid membrane. PC-SSLBs, which are largely driven toward the plus end of the microtubule, may also be used to investigate the Unc104 kinesin and possibly other *Dictyostelium* kinesins (Table S1). Note that Unc104 recruitment is insensitive to PA, whereas dynein-dynactin recruitment depends on PA (Figures 2E and 2F). Therefore, it should be possible in the future to tune the dynein:kinesin ratio by titrating the PC:PA ratio on SSLBs. Such experiments can help to understand how these opposing motors compete when they drive a cargo.[33]

The source matters: PA enhances kinesin-1-driven SSLB motion with rat brain ATP-releasate

Keeping in mind the well-established functions of PA in cell signaling,[12] vesicular trafficking,[10,11,13,14] and physiology/lipid homeostasis,[11,15] we next probed PA-motor interactions in an evolutionarily distant system, the mammalian brain. ATP-releasate was prepared from rat brain and incubated with PC or PA-SSLBs. Figure 3A shows representative video tracks of the free motion (optical trap switched off) for plus and minus-directed SSLBs. The motion of PC and PA-SSLBs can also be seen in Videos S3 and S4, respectively. Video tracks were parsed as described earlier to obtain the distribution of segment velocities. The mean velocity of minus-directed segments was similar for PA and PC-SSLBs, and the mean velocity of plus-directed segments on PA-SSLBs was also similar (Figure 3B).

We next moved to optical trapping of SSLBs on polarity-labeled MTs. Figure 3C shows representative data for a minus-directed PC-SSLB, a plus-directed PA-SSLB, and also a minus-directed PA-SSLB. We separate the observed motion into four categories, depicted as a bar graph (Figure 3D). Note that 42% of PC-SSLBs are non-motile, but this fraction is only 10% for PA-SSLBs. PA causes this improvement in overall motility by increasing the plus-directed fraction of SSLBs (8% for PC but 42% for PA-SSLBs). However, PA had no effect on minus-directed SSLB motion (42% for PC and 43% for PA-SSLBs), a finding that contrasts with the PA-induced improvement of minus-directed motion for *Dictyostelium*-SSLBs. PA therefore improves overall SSLB motility for both rat brain releasate and *Dictyostelium*, but this improvement has different mechanisms. For *Dictyostelium* minus-directed (dynein-driven) motion is upregulated, whereas for rat brain plus-directed (kinesin driven) motion is upregulated. An obvious explanation for this difference could be that more kinesin is recruited from rat brain releasate to PA-SSLBs. Indeed, SSLB pull-downs showed a small but consistent increase of kinesin-1 on PA-SSLBs (Figures 3E and 3F). A lipid blot supported the possibility that kinesin-1 in rat brain releasate has higher affinity for PA (Figure 3G).

Dynein was recruited equally to PC and PA-SSLBs (Figures 3E and 3F), consistent with the equal fraction of minus-moving PC and PA-SSLBs (Figure 3D). We have earlier found that PA has no detectable affinity for kinesin-2 or dynein from mammalian cell lysate.[11] Taken together, the enhanced plus-directed motion for PA-SSLBs with rat brain releasate likely derives from the higher abundance of kinesin-1 on PA-SSLBs. Further, dynactin was not detected by western blotting in rat brain ATP releasate (Figure S2B), an observation that agrees with others.[34] Accordingly, dynactin was also barely detectable on PC or PA-SSLBs prepared from rat brain releasate (Figure 3E). Note that multiple dynein motors can drive robust *in vitro* bead motion without dynactin,[27] and dynein functions during cell division may not require dynactin.[35] It is therefore important to study only dynein function on a membrane and also how the addition of dynactin can modulate such dynein function.[21] The PA-SSLB system using rat brain releasate shows ~43% minus-directed motion (Figure 3D) with very little dynactin (Figure 3E) and could thus help in this direction.

Distinct effects of kinesin-1 and dynein inhibition on SSLBs prepared using rat brain ATP releasate

PA-SSLBs incubated with rat brain ATP releasate exhibited equal probability of plus and minus motion (Figure 3D). We therefore realized that PA-SSLBs are an excellent experimental system to understand the controversial effects that inhibitors of dynein or kinesin-1 have on intracellular transport. Inhibiting one kind of motor may or may not allow the opposing motor to take over, depending on the "tug-of-war" versus "coordination" models of bidirectional transport.[33,36–38] To specifically inhibit kinesin-1 on PA-SSLBs, we used a GST-tagged kinesin tail domain peptide (KTD-GST) purified from bacteria.[11] KTD-GST contains the C-terminal end of the kinesin-1 heavy chain (amino acid 854 to 963), and it is known to inhibit kinesin-1 ATPase activity.[39–41] Only 10% of GST-control treated PA-SSLBs were non-motile; however, KTD-GST used at 170 nM concentration drastically increased this non-motile fraction to 81% (Figure 4A).

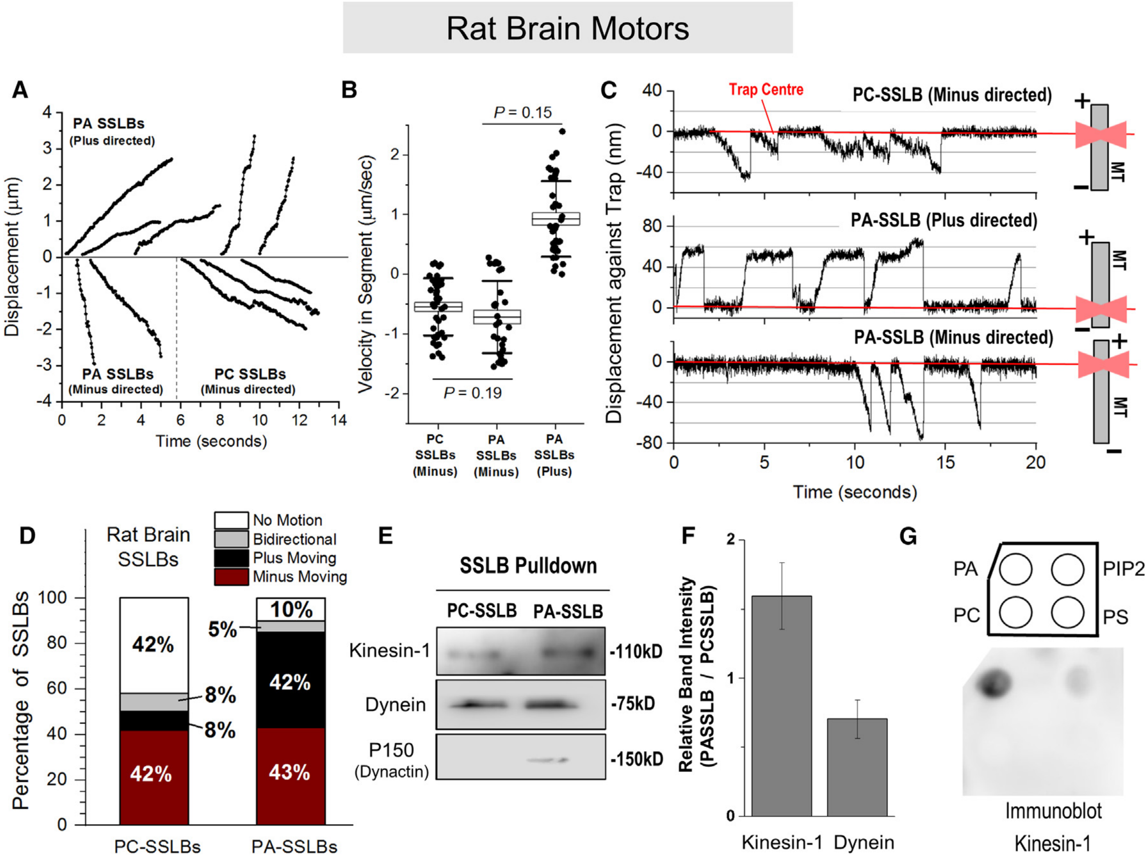

Figure 3. Phosphatidic acid enhances plus-directed motion of SSLBs prepared with rat brain ATP releasate

(A) Representative tracks for free runs of PC and PA-SSLBs shown as a displacement versus time graph. Tracks have been offset by arbitrary amounts for ease of representation.

(B) Boxplot representing the velocity within constant-velocity segments for PC and PA-SSLBs (see main text). Error bars represent SD, and boxes represent SEM. The *p* value is obtained using Student's t test by comparing the magnitude of the mean of segment velocities (direction of motion was ignored during comparison).

(C) Displacement versus time curves for the motion of PC and PA-SSLBs against an optical trap. Examples of plus as well as minus-directed motion are shown for PA-SSLBs. Schematics on the right depict the experimental geometry and MT polarity. Red lines indicate the center of the trap (zero position). The trap stiffness is 0.07 ± 0.01 pN/nm.

(D) Categorization of SSLBs based on their motion on polarity-labeled MTs. PA-SSLBs have a small fraction of immotile SSLBs (10%) compared with PC-SSLBs (42%). Note the increase of plus-moving SSLBs upon inclusion of PA. A total of 44 and 62 PC-SSLBs and PA-SSLBs were analyzed, respectively.

(E) Immunoblots representing motors retained on SSLBs in an SSLB pull-down assay carried out from the rat motor pool. SSLBs were incubated with rat brain ATP releasate, washed, and probed for bound/retained motors. PC and PA-SSLB numbers were normalized by optical density.

(F) Quantitation of motors bound to PC and PA-SSLBs in the SSLB pull-down assay. Error bars are mean ± SEM, $N = 3$.

(G) Lipid blot demonstrating the ability of negatively charged lipids to recruit kinesin-1 from the ATP releasate fraction. Phosphatidic acid (PA) shows strong binding of kinesin-1. Phosphatidylinositol-4,5-bisphosphate (PIP_2) shows weak recruitment of kinesin-1. Phosphatidylserine (PS) and phosphatidylcholine (PC) show no detectable binding.

To cause an 81% block in motion, KTD-GST must somehow be able to inhibit motion of kinesin-driven (42% in GST-control) as well as dynein-driven PA-SSLBs (43% in GST-control). KTD removed neither kinesin-1 nor dynein from PA-SSLBs (Figures 4B and 4C). KTD is not a generic inhibitor of motors, as it had no effect on motion of PA-SSLBs prepared from *Dictyostelium* releasate that lacks kinesin-1 (Figure S2C). Thus, KTD must be able to also block dynein when it inhibits kinesin-1 on PA-SSLBs. Coy et al.[41] found that KTD reduces the velocity of MT-gliding by kinesin-1 and thus suggested that KTD reduces kinesin's OFF-rate from the MT; i.e., it causes kinesin to bind tightly to the MT.[39–41] Very interestingly, for the SSLBs whose motion was inhibited by KTD, we could never pull away the SSLB from the MT using the optical trap. It appeared as if these SSLBs were tightly bound/locked to the MT. Further, KTD marginally increased the amount of kinesin-1 (but not of dynein) pelleting with MTs (Figures 4D and 4E). This assay was done in presence of ATP (~1 mM) to resemble the motility experiment. Taken together, it appears that KTD blocks kinesin as well as dynein-driven SSLB motion because it causes kinesin-1 to bind more tightly (i.e., to get "locked") to the MT.

We next used ciliobrevin D, a well-known inhibitor of dynein,[42] on PA-SSLBs prepared from rat brain ATP releasate. Ciliobrevin

CellPress
OPEN ACCESS

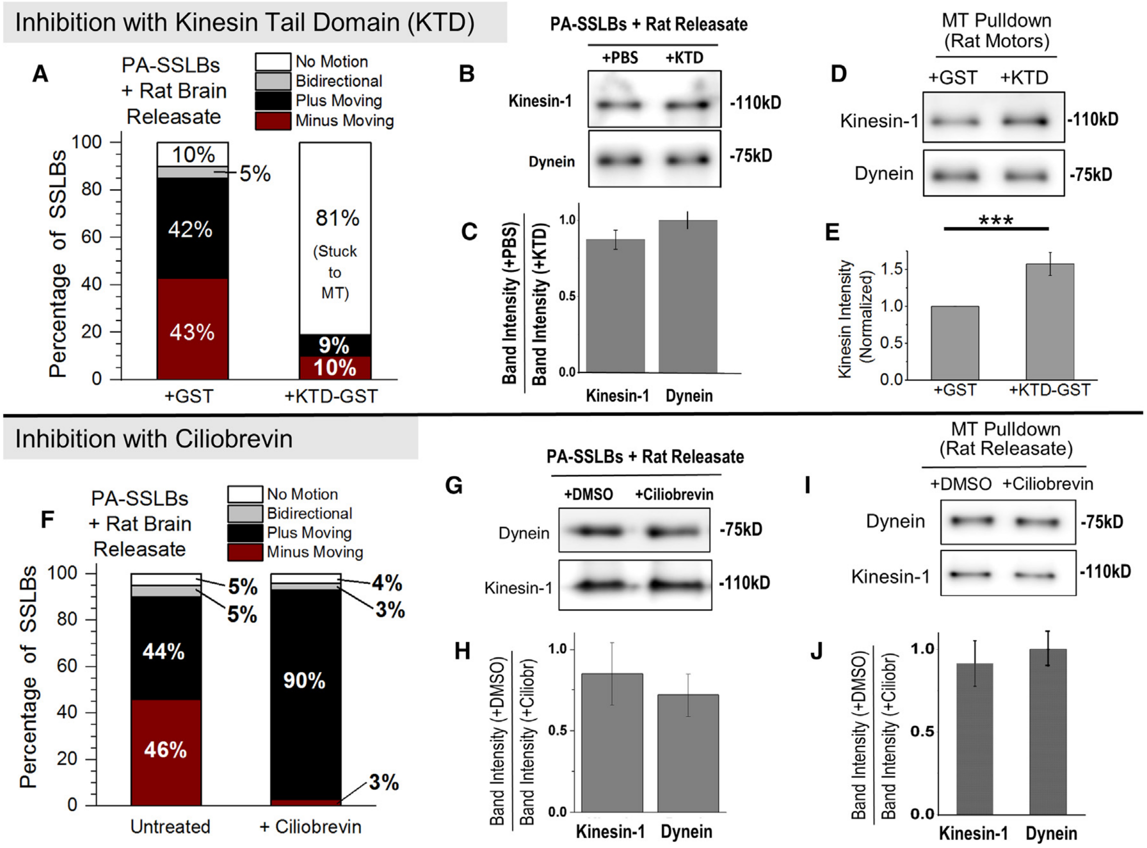

Figure 4. Inhibition of SSLB motion prepared from rat brain ATP releasate using kinesin tail domain and ciliobrevin

(A) Categorization of PA-SSLBs incubated with rat brain releasate in presence of 170 nM GST (control) or 170 nM KTD-GST. KTD implies "kinesin-1 tail domain." A total of 30 and 47 SSLBs were analyzed for GST and KTD-GST, respectively. KTD-GST induces most PA-SSLBs (81%) to get stuck on MTs. Once stuck, these SSLBs cannot be removed from the MT by the trap.

(B) Immunoblots representing proteins retained on PA-SSLBs during SSLB pull-down assay. PA-SSLBs were incubated with rat brain motor fractions along with PBS (as a control) or 170 nM KTD-GST, washed, and then probed for kinesin-1 and dynein.

(C) Quantitation of kinesin-1 and dynein intensity in the SSLB pull-down assay. No significant depletion of kinesin-1 occurs from the SSLBs upon KTD treatment. $N = 3$. Data represent mean ± SEM, $p > 0.05$.

(D) Immunoblot depicting kinesin-1 and dynein bound to microtubules after a microtubule-pelleting assay was performed in presence of ATP releasate from rat brain. MT pull-down was done in presence of ATP to mimic the conditions in motility assay. GST peptide (control) or KTD-GST was present at 170 nM concentration during the MT pull-down.

(E) Quantification of kinesin-1 bound to microtubule pellet. Kinesin-1 shows significantly higher binding in presence of KTD-GST compared to the GST-control. $N = 6$. Data represent mean ± SEM. $^{***}p < 0.001$. The p value is obtained using Student's t test.

(F) Categorization of PA-SSLBs incubated with rat brain releasate, based on their motion on polarity-labeled MTs in the presence and absence of ciliobrevin. The motile fraction of PA-SSLBs treated with 20 μM ciliobrevin remains unchanged (~95% in each case). Ciliobrevin inhibits minus-directed motion. PA-SSLB motion in the presence of ciliobrevin is almost completely plus-directed driven by kinesin. 130 and 18 SSLBs were analyzed in ciliobrevin and DMSO groups, respectively. $N = 3$.

(G) Immunoblots representing proteins retained on PA-SSLBs after SSLB pull-down. PA-SSLBs were incubated with rat brain motor releasate in the presence of 20 μM ciliobrevin or DMSO, washed, and probed for bound/retained proteins.

(H) Quantitation of band intensity of motors in the SSLB pull-down assay. No significant depletion of motors is seen on SSLBs after ciliobrevin treatment. $N = 3$. Data represent mean ± SEM.

(I) Immunoblot depicting kinesin-1 and dynein bound to microtubules after a microtubule-pelleting assay performed in the presence of ATP releasate from rat brain. Experiment was done in the presence of ATP to mimic the conditions in motility assay. Lanes represent data where DMSO (control) or 20 μM ciliobrevin was added.

(J) Quantitation of kinesin-1 and dynein amounts bound to microtubules in the presence of 20 μM ciliobrevin or DMSO in MT pelleting assay. Data represent mean ± SEM, n.s. represents $p > 0.05$, $N = 3$.

may inhibit dynein ATPase activity by acting as a competitor to nucleotides,[43] but the exact mechanism of inhibition is not known. Ciliobrevin used at 20 μM expectedly reduced the fraction of minus-directed PA-SSLBs (Figure 4F). However, ciliobrevin-treated PA-SSLBs exhibited a surprising and dramatic compensatory increase in plus-directed motion, so that their overall motile fraction was comparable to untreated PA-SSLBs (~90%; Figure 4F). Ciliobrevin obviously did not inhibit kinesin-1 because

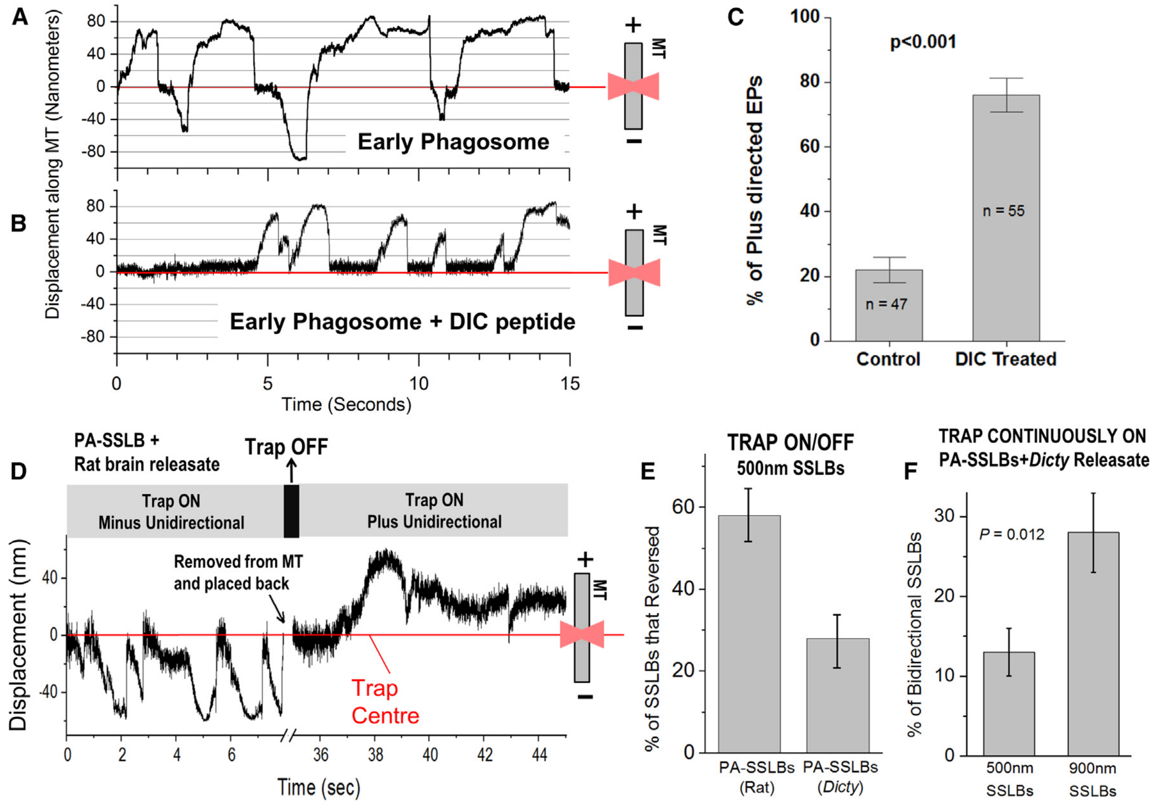

Figure 5. Engagement of opposite polarity motors on SSLBs

(A) Displacement-time graph representing motion against the optical trap of an “early phagosome” (EP) isolated from *Dictyostelium*. Unlike rarely observed bidirectional motion in SSLBs, EPs show rapid and consecutive stalls in opposite directions.

(B) A representative displacement vs. time graph for an EP treated with dynein intermediate chain (DIC) peptide present at 0.25 μM concentration. The EP converts from bidirectional (see A) to unidirectional plus end driven after addition of DIC.

(C) Bar graph representing the percentage of plus-end-directed EPs in control (untreated) and DIC-treated early phagosomes. Error bars are SEM. *p* value is obtained using Student’s t test.

(D) Displacement versus time against an optical trap for a PA-SSLB prepared from rat brain releasate. The SSLB initially shows dynein-driven (minus-directed) motion on a polarity-labeled MT. The SSLB is removed from the MT at the time point indicated and then placed back on the MT after a few seconds. This separation from the MT may allow rotational diffusion of the SSLB. Direction of motion reverses when the SSLB is placed back on the MT, presumably because a new area of the SSLB (now containing kinesin-1 motors) is now proximal to the MT.

(E) Fraction of PA-SSLBs from *Dictyostelium* or from rat brain releasate that reversed direction in a trap ON/OFF experiment (see Figure 5D). Error bars are SEM, *N* = 3. *p* value is obtained using Student’s t test.

(F) Fraction of PA-SSLBs (diameter 500 nm or 900 nm) driven by *Dictyostelium* motors that reverse direction on the MT when moving against an optical trap that is continuously ON. Error bars are SD. *N* = 3. *p* value is obtained using Student’s t test.

ciliobrevin-treated PA-SSLBs displayed abundant plus-directed motion. Earlier reports have demonstrated that kinesin-1 activity is unaffected up to 100 μM ciliobrevin.[43] Ciliobrevin did not remove dynein or kinesin-1 from PA-SSLBs, as determined by an SSLB pull-down experiment (Figures 4G and 4H). Unlike the effect of KTD, ciliobrevin-treated PA-SSLBs could be pulled off easily from MTs by the trap. Ciliobrevin also had no effect on kinesin-1 or dynein pelleting with MTs in presence of 1 mM ATP (Figures 4I and 4J), suggesting that unlike KTD, ciliobrevin is not a “locking inhibitor” that causes dynein to bind tightly to MTs at physiological ATP concentration (~1 mM). Based on these observations, we believe that kinesin-1 can overcome the ciliobrevin-inactivated dyneins to reduce minus-directed motion and to cause overwhelmingly plus-directed motion of PA-SSLBs (Figure 4F). This compensatory increase of plus-directed motion by ciliobrevin (Figure 4F) contrasts with the overall arrest of PA-SSLB motion after KTD treatment (Figure 4A).

The above data suggest that different kinds of inhibitors of motors have contrasting effects on SSLB motion, depending on the exact mechanism of inactivation. We next tested this possibility for a native-like cellular cargo, namely early phagosomes (EPs) that result from phagocytosing latex beads into *Dictyostelium* cells. EPs show rapid alternating activity of dynein and kinesin against an optical trap inside cells, and the same behavior is reproduced for purified EPs moving along MTs *in vitro.*[44] During such “bidirectional” stalls, once a given class of motors on the EP has detached, the opposing motors often generate force within ~1 s (see Figure 5A). To inhibit dynein on EPs, we used a small peptide consisting of the N-terminal region (1–126 amino acids) of *Dictyostelium* dynein intermediate chain (DIC). This

CellPress
OPEN ACCESS

peptide blocks the interaction between dynactin and endogenous DIC within the dynein-dynactin complex. We have shown that this peptide is a specific inhibitor of dynein-driven transport of late phagosomes in *Dictyostelium*,[21] a finding in good agreement with several studies on the mammalian version of this peptide.[31,32] DIC-peptide used at 0.25 μM converted the bidirectional motion of EPs to plus-directed unidirectional motion, as shown for a representative EP (Figure 5B). This conversion is quantified for a large population of EPs, showing that the overwhelming majority of DIC-treated EPs show plus-directed motion (Figure 5C). Therefore, DIC and ciliobrevin inhibit dynein in a "non-locking" manner because kinesin can now drive SSLB motion, whereas KTD "locks" kinesin to block both kinesin and dynein-driven motion.

Unidirectional motion of SSLBs and implications to intracellular transport

Similar to EPs, many kinds of vesicles move in a bidirectional (back-and-forth) manner along MTs inside cells[33,36] and *in vitro*.[17,37,38] We were therefore very surprised that a particular SSLB would almost always move in the same direction (plus or minus) in our optical trap assay. This rarity of bidirectional motion was true for all combinations of lipids and motors on SSLBs (Figures 2D and 3D). The "unidirectional" nature of SSLBs was most intriguing for PA-SSLBs driven by rat brain ATP releasate, because as a population, these SSLBs showed an equal amount of plus and minus motion (Figure 3D). Both plus and minus motors therefore have almost equal activity on PA-SSLBs. However, once a given type of motor attaches to MT from the SSLB, it continues to attach/detach/re-attach, whereas the other type of motor is not able to engage. Why are reversals so rare for SSLBs, and might this provide interesting clues about regulation of intracellular transport? We note that lipid droplets purified from COS-1 cells stall repeatedly against the trap in only one direction that could be plus or minus,[45] similar to our observation on PA-SSLBs. It is therefore interesting to probe this behavior further.

A possible but unlikely explanation for unidirectional PA-SSLBs is that some unknown mechanism causes plus and minus motors to segregate away onto two separate SSLB populations. A given PA-SSLB with minus-directed motors may then have almost no plus-directed motors and vice versa, thus yielding unidirectional motion for each population. We tested this unlikely possibility using an experiment schematized in Figure 5D. We allowed a PA-SSLB prepared from rat brain releasate to generate force and then removed the trapped SSLB away from the MT and the cover slip. We now switched the trap OFF to allow the SSLB to diffuse/rotate, switched the trap ON to again trap the same SSLB, and then lowered the SSLB onto the same MT for motor re-attachment. As shown in Figure 5E, ~55% of such re-attached PA-SSLBs now "reversed" to generate force only in the opposite direction (see the example in Figure 5D). The remaining SSLBs continued to generate force only in the original direction. This finding is in good agreement with the equal fraction of plus- and minus-moving PA-SSLBs in presence of rat brain ATP releasate (Figure 3D). Thus, a given PA-SSLB incubated with rat brain ATP releasate has equal activity of both kinds of motors, but once one type of motor engages, the other type rarely gets a chance. Does this situation hold true for PA-SSLBs prepared using *Dictyostelium* ATP releasate? Such SSLBs showed only ~25% chance of reversing after they were re-attached to the MT (Figure 5E). This is exactly what one would predict because only a minor fraction of the *Dictyostelium* PA-SSLBs show kinesin activity (15% + 12% = 27%), with most of them being minus-directed (~64%; Figure 2D).

We have earlier derived an expression for the approximate contact area on a spherical cargo (= $A_{CONTACT}$), from where a motor of given size can physically reach a MT.[44] The expression for $A_{CONTACT}$ is shown and the relevant terms are explained in Figure S2D, where cargoes of 500- and 900-nm diameter are also drawn to relative scale. Each cargo is attached to an MT at its base via individual motors (~70-nm length). Only those motors that reside within $A_{CONTACT}$ (black patch) are able to reach the MT. Figure S2D also depicts the calculated dependence of $A_{CONTACT}$ on cargo size. The 900-nm SSLB has larger $A_{CONTACT}$. If the surface density of membrane-bound motors is same for 500-nm and 900-nm SSLBs, then the probability that plus and minus motors can both contact an MT at the same time should increase for larger SSLBs. We therefore prepared PA-SSLBs using 500-nm-diameter (our original size) and larger 900-nm-diameter beads, incubated both with *Dictyostelium* ATP releasate, and we tested motion when these were held in the optical trap. Indeed, 900-nm PA-SSLBs displayed more bidirectional events compared to 500-nm PA-SSLBs (Figure 5F). Taken together, these findings imply that larger cargoes could be more prone to reversals during motion.

DISCUSSION

SSLBs provide a simple method to precisely measure the activity of microtubule motors on a cargo bound by a lipid membrane. This assay is unique because it allows optical-trap-based measurement of motor protein force (i.e., activity) at very small concentrations of rare lipids, which is physiologically relevant for these lipids. As an example, we have measured activity of motors on membranes containing only 5 mol % of PA. Such biophysical measurements using SSLBs may help dissect how rare lipids activate motors to generate polarized distributions of vesicles inside cells for downstream cellular requirements. Note that higher force can arise because more motors are present on a particular kind of SSLB and/or also because of the local geometry of lipid-motor binding.[2] While the first possibility can be investigated using biochemical assays (for example, see Figure 2E), the exact contribution of lipid-motor geometry on SSLBs to force generation is harder to dissect. We did find, however, that other acidic phospholipids (PS and PIP_2) bind dynein with much lower affinity than PA (Figure 2G). Therefore, in addition to its negative charge, the conical shape of PA may also aid dynein-dynactin binding. PA-SSLBs incubated with *Dictyostelium* releasate have more dynein-dynactin and show robust, long-distance minus-directed motion. Perhaps the conical shape of PA generates negative curvature to locally raise the SSLB membrane,[14] and this allows PA-bound dyneins to access the MT more easily and generate higher force. However, these possibilities are speculative at

present, requiring verification using experiments and/or computational approaches.

Because PA-SSLBs prepared with rat brain releasate exhibit equal probability of plus and minus motion, we used this system to understand how inhibiting a specific motor can affect overall cargo motion. Very interestingly, KTD (a kinesin-1 inhibitor) reduced both kinesin-1 and dynein-driven motion, whereas ciliobrevin or DIC blocked only dynein with a compensatory increase in plus-directed motion. KTD-treated non-motile SSLBs could not be pulled away from the MT by the trap. We therefore believe that KTD blocked the ATPase activity of kinesin-1 causing SSLBs to get "locked" onto MTs and thus also blocking dynein-driven motion. However, ciliobrevin and DIC inactivated dynein without a locking effect, and kinesin-1 could now drive motion. Thus, KTD and ciliobrevin/DIC have contrasting effects. This finding has specific implications to the "paradox of co-dependence," wherein inhibiting one motor-type enhances motion in the opposite direction in some instances, but it can also completely abrogate motion in other instances. This paradox has been discussed in detail, and numerous inhibition-based studies have been cited in a review article by Hancock.[33] Based on our results with SSLBs, the paradox can be explained if motor inhibitors can be classified as "locking inhibitors" (e.g., KTD) or "non-locking inhibitors" (e.g., ciliobrevin and DIC).

PA-SSLBs incubated with rat motors showed motion in both directions, but a given SSLB rarely reversed direction. This may happen if the moving SSLB cannot rotate as it is bound to the MT by motor(s) and also because the surface (e.g., cover slip) increases the rotational drag on the SSLB. Increasing SSLB size (and therefore $A_{CONTACT}$) promoted reversals (Figure 5F), likely because it increased the chance that both kinds of motors bind the MT at the same time or in quick succession. Thus, a larger cargo may be able to back out and resume motion in a different direction when facing obstructions in the crowded cytoskeletal space. The reported diffusion constant for kinesin-1 in a membrane is D_{LIPID} = 1.4 $\mu m^2/s$.[5] The residence time of a diffusing kinesin inside $A_{CONTACT}$ on a 500-nm SSLB (= 0.063 μm^2) is T_{RES} = 0.063/[4×D_{LIPID}] = 11 ms. Depending on the geometry of interaction, kinesin-1 requires between 200 and 1400 ms to bind to an MT.[46] If we assume an average time to binding of 800 ms, then a single kinesin diffusing along on the SSLB membrane has a probability of only 11/800 (≈1%) of binding to the MT before it diffuses away out of $A_{CONTACT}$. In short, most kinesin (and presumably dynein) motors diffuse in and out of $A_{CONTACT}$ without ever binding to the MT. With the above in mind, two points become relevant to an SSLB that is moving unidirectionally. First, a new opposing motor (kinesin or dynein) can rarely engage due to its rapid diffusion in the membrane and because already bound motors restrict free rotation of the cargo. Second, based on force measurements, these cargoes are usually driven by 5–8 dyneins and 1–2 kinesin motors,[21,37,44] consistent with ~6-fold more dynein compared to kinesin-1 on neuronal vesicles.[38] A kinesin-driven SSLB would require 4–5 dyneins to bind the MT simultaneously before these dyneins can overpower the stronger kinesin to reverse the direction. Even if a dynein diffusing into $A_{CONTACT}$ manages to bind the MT, it would often get detached by the opposing high force from kinesin. For a multiple-dynein-driven SSLB, the lower abundance of kinesin-1 implies a longer wait time before a kinesin-1 can bind the MT and reverse the motion.

We emphasize that both bidirectional and unidirectional motion of cargoes has been reported.[33,36] *In vitro*, early endosomes and phagosomes exhibit bidirectional motion.[37,44] Liposome-bound motors are bidirectional even without regulatory proteins.[17] However, late phagosomes are unidirectional inside cells as well as *in vitro*.[47] Lipid droplets purified from rat liver[16] and from COS-1 cells[45] also exhibit unidirectional stalls like the SSLBs reported here. It is therefore plausible that some cellular cargoes are bidirectional, whereas others are unidirectional by design. Specific lipids and membrane-bound proteins could ensure that kinesin and dynein are both present within small clusters on the membrane of bidirectional cargoes. Regulatory proteins could also modulate ON or OFF rates of motors to the MT, so that kinesin and dynein can bind the MT rapidly to generate bidirectional motion. Populating a specific motor type and excluding the other within a cluster could also make the cargo unidirectional, as we have observed for dynein on late phagosomes.[9] Predictions from computational studies of motor organization/diffusion on membranes are crucial, and we hope that the promising results on kinesin[48] can be expanded to study cargoes with kinesin and dynein.

Limitations of the study

We rarely observed bidirectional SSLBs, likely because our two-component lipid system (PC and PA) did not replicate the structure of complex cellular membranes. The dynein:kinesin ratio on SSLBs could also be different from cellular membranes, and certain regulatory proteins that modulate motor-microtubule ON/OFF rates could be absent from SSLBs. In order to obtain bidirectional SSLBs, a more native-like lipid mixture might be required. The aforesaid clusters of membrane-bound motors, if at all they exist, would be difficult to visualize on the curved surface of SSLBs. Such visualization might be easier on planar SLBs if super-resolution techniques are employed.

STAR★METHODS

Detailed methods are provided in the online version of this paper and include the following:

- KEY RESOURCES TABLE
- RESOURCE AVAILABILITY
  - Lead contact
  - Materials availability
  - Data and code availability
- EXPERIMENTAL MODEL AND STUDY PARTICIPANT DETAILS
  - Animals and *Dictyostelium*
- METHOD DETAILS
  - Liposome and SSLB preparation
  - In-gel protein digestion, peptide extraction and mass spectrometry analysis
- QUANTIFICATION AND STATISTICAL ANALYSIS

SUPPLEMENTAL INFORMATION

Supplemental information can be found online at https://doi.org/10.1016/j.celrep.2024.114252.

ACKNOWLEDGMENTS

We thank the Sophisticated Analytical Instrument Facility (SAIF) at Indian Institute of Technology Bombay for use of the Q-Exactive Plus Biopharma-High Resolution Orbitrap Liquid Chromatograph Mass Spectrometer. We thank the Tata Institute of Fundamental Research, Dr. Vidita Vaidya, and Dr. Shital Suryavanshi for access to their animal facility. This work was supported by an Augmented Seed Grant of the Indian Institute of Technology Bombay and from the Department of Biotechnology – Wellcome Trust India Alliance Senior Fellowships to R.M. (grants WT079214MA, IA/S/11/2500255, and IA/S/19/2/504634). P.K. acknowledges support from the S.P. Mukherjee Fellowship of the Council for Scientific and Industrial Research (CSIR), Government of India. P.S. acknowledges funding from a Department of Biotechnology – Wellcome Trust India alliance Early Career Fellowship (grant IA/E/15/1/502298).

AUTHOR CONTRIBUTIONS

P.K., D.C., P.S., and A.M. designed and conducted experiments under supervision from R.M. All authors contributed to writing the paper.

DECLARATION OF INTERESTS

The authors declare no competing interests.

Received: December 2, 2023
Revised: April 1, 2024
Accepted: May 3, 2024
Published: May 20, 2024

REFERENCES

1. Vale, R.D. (2003). The molecular motor toolbox for intracellular transport. Cell *112*, 467–480.
2. Pathak, D., and Mallik, R. (2017). Lipid - Motor Interactions: Soap Opera or Symphony? Curr. Opin. Cell Biol. *44*, 79–85.
3. Cook, N.R., and Davidson, H.W. (2001). In vitro assays of vesicular transport. Traffic *2*, 19–25.
4. Klopfenstein, D.R., Tomishige, M., Stuurman, N., and Vale, R.D. (2002). Role of phosphatidylinositol(4,5)bisphosphate organization in membrane transport by the Unc104 kinesin motor. Cell *109*, 347–358.
5. Grover, R., Fischer, J., Schwarz, F.W., Walter, W.J., Schwille, P., and Diez, S. (2016). Transport efficiency of membrane-anchored kinesin-1 motors depends on motor density and diffusivity. Proc. Natl. Acad. Sci. *113*, e7185–e7193. https://doi.org/10.1073/pnas.1611398113.
6. Nelson, S.R., Trybus, K.M., and Warshaw, D.M. (2014). Motor coupling through lipid membranes enhances transport velocities for ensembles of myosin Va. Proc. Natl. Acad. Sci. USA *111*, E3986–E3995.
7. Li, Q., Tseng, K.F., King, S.J., Qiu, W., and Xu, J. (2018). A fluid membrane enhances the velocity of cargo transport by small teams of kinesin-1. J. Chem. Phys. *148*, 123318.
8. Wassmer, T., Attar, N., Harterink, M., van Weering, J.R.T., Traer, C.J., Oakley, J., Goud, B., Stephens, D.J., Verkade, P., Korswagen, H.C., and Cullen, P.J. (2009). The Retromer Coat Complex Coordinates Endosomal Sorting and Dynein-Mediated Transport, with Carrier Recognition by the trans-Golgi Network. Dev. Cell *17*, 110–122.
9. Rai, A., Pathak, D., Thakur, S., Singh, S., Dubey, A.K., and Mallik, R. (2016). Dynein Clusters into Lipid Microdomains on Phagosomes to Drive Rapid Transport toward Lysosomes. Cell *164*, 722–734.
10. Wang, Z., Zhang, F., He, J., Wu, P., Tay, L.W.R., Cai, M., Nian, W., Weng, Y., Qin, L., Chang, J.T., et al. (2017). Binding of PLD2-Generated Phosphatidic Acid to KIF5B Promotes MT1-MMP Surface Trafficking and Lung Metastasis of Mouse Breast Cancer Cells. Dev. Cell *43*, 186–197.e7. https://doi.org/10.1016/j.devcel.2017.09.012.
11. Kumar, M., Ojha, S., Rai, P., Joshi, A., Kamat, S.S., and Mallik, R. (2019). Insulin activates Intracellular transport of Lipid Droplets to release Triglycerides from the Liver. J. Cell Biol. *218*, 3697–3713. https://doi.org/10.1083/jcb.201903102.
12. Shin, J.J.H., and Loewen, C.J.R. (2011). Putting the pH into phosphatidic acid signaling. BMC Biol. *9*, 85.
13. Thakur, R., Naik, A., Panda, A., and Raghu, P. (2019). Regulation of membrane turnover by phosphatidic acid: Cellular functions and disease implications. Front. Cell Dev. Biol. *7*, 83. https://doi.org/10.3389/fcell.2019.00083.
14. Zhukovsky, M.A., Filograna, A., Luini, A., Corda, D., and Valente, C. (2019). Phosphatidic acid in membrane rearrangements. FEBS Lett. *593*, 2428–2451.
15. Athenstaedt, K., and Daum, G. (1999). Phosphatidic acid, a key intermediate in lipid metabolism. Eur. J. Biochem. *266*, 1–16. https://doi.org/10.1046/j.1432-1327.1999.00822.x.
16. Rai, P., Kumar, M., Sharma, G., Barak, P., Das, S., Kamat, S.S., and Mallik, R. (2017). Kinesin-dependent mechanism for controlling triglyceride secretion from the liver. Proc. Natl. Acad. Sci. USA *114*, 12958–12963.
17. D'Souza, A.I., Grover, R., Monzon, G.A., Santen, L., and Diez, S. (2023). Vesicles driven by dynein and kinesin exhibit directional reversals without regulators. Nat. Commun. *14*, 7532.
18. Reck-Peterson, S.L., Redwine, W.B., Vale, R.D., and Carter, A.P. (2018). The cytoplasmic dynein transport machinery and its many cargoes. Nat. Rev. Mol. Cell Biol. *19*, 382–398. https://doi.org/10.1038/s41580-018-0004-3.
19. Bingham, J.B., King, S.J., and Schroer, T.A. (1998). Purification of dynactin and dynein from brain tissue. Methods Enzymol. *298*, 171–184.
20. D'Souza, A., Sanghavi, P., Rai, A., Pathak, D., and Mallik, R. (2016). Isolation of Latex Bead Phagosomes from Dictyostelium for in vitro Functional Assays. Bio. Protoc. *6*, e2056.
21. Sanghavi, P., Kumar, P., Roy, A., Madhusudhan, M.S., and Mallik, R. (2021). On and off controls within dynein-dynactin on native cargoes. Proc. Natl. Acad. Sci. USA *118*, e2103383118.
22. Blocker, A., Severin, F.F., Burkhardt, J.K., Bingham, J.B., Yu, H., Olivo, J.C., Schroer, T.A., Hyman, A.A., and Griffiths, G. (1997). Molecular requirements for bi-directional movement of phagosomes along microtubules. J. Cell Biol. *137*, 113–129.
23. Barak, P., Rai, A., Rai, P., and Mallik, R. (2013). Quantitative optical trapping on single organelles in cell extract. Nat. Methods *10*, 68–70.
24. Soppina, V., Rai, A., and Mallik, R. (2009). Simple non-fluorescent polarity labeling of microtubules for molecular motor assays. Biotechniques *46*, 543–549.
25. Zinser, E., Sperka-Gottlieb, C.D., Fasch, E.V., Kohlwein, S.D., Paltauf, F., and Daum, G. (1991). Phospholipid synthesis and lipid composition of subcellular membranes in the unicellular eukaryote Saccharomyces cerevisiae. J. Bacteriol. *173*, 2026–2034.
26. Petrov, D.Y., Mallik, R., Shubeita, G.T., Vershinin, M., Gross, S.P., and Yu, C.C. (2007). Studying molecular motor-based cargo transport: what is real and what is noise? Biophys. J. *92*, 2953–2963.
27. Mallik, R., Petrov, D., Lex, S.A., King, S.J., and Gross, S.P. (2005). Building Complexity: An In Vitro Study of Cytoplasmic Dynein with In Vivo Implications. Curr. Biol. *15*, 2075–2085.
28. Koonce, M.P. (2020). 13 Plus 1: A 30-Year Perspective on Microtubule-Based Motility in Dictyostelium. Cells *9*, 528.
29. Li, D. (2022). Role of Spectrin in Endocytosis. Cells *11*, 2459.
30. Muresan, V., Stankewich, M.C., Steffen, W., Morrow, J.S., Holzbaur, E.L., and Schnapp, B.J. (2001). Dynactin-dependent, dynein-driven vesicle transport in the absence of membrane proteins: A role for spectrin and acidic phospholipids. Mol. Cell *7*, 173–183.
31. Vaughan, P.S., Leszyk, J.D., and Vaughan, K.T. (2001). Cytoplasmic Dynein Intermediate Chain Phosphorylation Regulates Binding to Dynactin. J. Biol. Chem. *276*, 26171–26179.

32. Towns, W.L., Tauhata, S.B.F., Vaughan, P.S., and Vaughan, K.T. (2009). Transfection-induced defects in dynein-driven transport: Evidence that ICs mediate cargo-binding. Cell Motil Cytoskeleton *66*, 80–89.

33. Hancock, W.O. (2014). Bidirectional cargo transport: moving beyond tug of war. Nat. Rev. Mol. Cell Biol. *15*, 615–628.

34. Ori-McKenney, K.M., Xu, J., Gross, S.P., and Vallee, R.B. (2010). A cytoplasmic dynein tail mutation impairs motor processivity. Nat. Cell Biol. *12*, 1228–1234.

35. Wadsworth, P., and Lee, W.L. (2013). Microtubule motors: Doin' it without dynactin. Curr. Biol. *23*, R563–R565.

36. Welte, M.A. (2004). Bidirectional transport along microtubules. Curr. Biol. *14*, R525–R537.

37. Soppina, V., Rai, A.K., Ramaiya, A.J., Barak, P., and Mallik, R. (2009). Tug-of-war between dissimilar teams of microtubule motors regulates transport and fission of endosomes. Proc. Natl. Acad. Sci. USA *106*, 19381–19386.

38. Hendricks, A.G., Perlson, E., Ross, J.L., Schroeder, H.W., 3rd, Tokito, M., and Holzbaur, E.L.F. (2010). Motor Coordination via a Tug-of-War Mechanism Drives Bidirectional Vesicle Transport. Curr. Biol. *20*, 697–702. https://doi.org/10.1016/j.cub.2010.02.058.

39. Hackney, D.D., and Stock, M.F. (2000). Kinesin's IAK tail domain inhibits initial microtubule-stimulated ADP release. Nat. Cell Biol. *2*, 257–260.

40. Verhey, K.J., and Hammond, J.W. (2009). Traffic control: regulation of kinesin motors. Nat. Rev. Mol. Cell Biol. *10*, 765–777.

41. Coy, D.L., Hancock, W.O., Wagenbach, M., and Howard, J. (1999). Kinesin's tail domain is an inhibitory regulator of the motor domain. Nat. Cell Biol. *1*, 288–292.

42. Roossien, D.H., Miller, K.E., and Gallo, G. (2015). Ciliobrevins as tools for studying dynein motor function. Front. Cell. Neurosci. *9*, 252–310.

43. Firestone, A.J., Weinger, J.S., Maldonado, M., Barlan, K., Langston, L.D., O'Donnell, M., Gelfand, V.I., Kapoor, T.M., and Chen, J.K. (2012). Small-molecule inhibitors of the AAA+ ATPase motor cytoplasmic dynein. Nature *484*, 125–129.

44. Sanghavi, P., D'Souza, A., Rai, A., Rai, A., Padinhatheeri, R., and Mallik, R. (2018). Coin Tossing Explains the Activity of Opposing Microtubule Motors on Phagosomes. Curr. Biol. *28*, 1460–1466.e4.

45. Reddy, B.J.N., Mattson, M., Wynne, C.L., Vadpey, O., Durra, A., Chapman, D., Vallee, R.B., and Gross, S.P. (2016). Load-induced enhancement of Dynein force production by LIS1-NudE in vivo and in vitro. Nat. Commun. *7*, 12259–12314.

46. Janakaloti Narayanareddy, B.R., Allipeta, N.R., Allard, J., and Gross, S.P. (2024). A new method to experimentally quantify dynamics of initial protein–protein interactions. Commun. Biol. *7*, 311.

47. Rai, A.K., Rai, A., Ramaiya, A.J., Jha, R., and Mallik, R. (2013). Molecular adaptations allow dynein to generate large collective forces inside cells. Cell *152*, 172–182.

48. Bovyn, M., et al. (2021). Diffusion of Kinesin Motors on Cargo Can Enhance Binding and Run Lengths during Intracellular Transport. https://doi.org/10.5281/zenodo.4325111.

49. Perez-Riverol, Y., Bai, J., Bandla, C., García-Seisdedos, D., Hewapathirana, S., Kamatchinathan, S., Kundu, D.J., Prakash, A., Frericks-Zipper, A., Eisenacher, M., et al. (2022). The PRIDE database resources in 2022: A hub for mass spectrometry-based proteomics evidences. Nucleic Acids Res. *50*, D543–D552.

CellPress
OPEN ACCESS

STAR★METHODS

KEY RESOURCES TABLE

REAGENT or RESOURCE	SOURCE	IDENTIFIER
Antibodies		
Mammalian-DIC	SantaCruz	Sc13524
Kinesin-2	Abclonal	11259
P150	BD Biosciences	612709
Tubulin	Invitrogen	236-10501
GST	Cloud Clone	TAX158Ge22
Kinesin-1	Kumar et al.[11]	N/A
Dictyostelium dynein heavy chain	Soppina et al.[37]	N/A
Unc104	Soppina et al.[37]	N/A
Dictyostelium dynactin	Sanghavi et al.[44]	N/A
Plant actin	Abclonal	AC009
Chemicals, peptides, and recombinant proteins		
18:1(delta9-cis)-Phosphatidylcholine (DOPC)	Avanti Polar Lipids	850375P
3-sn-Phosphatidic acid (PA) from egg yolk	Avanti Polar Lipids	P9511
Phosphatidyl-inositol(4,5)bisphosphate (PIP_2)	Avanti Polar Lipids	850155P
18:1Liss Rhodamine-Phosphatidylethanolamine (Rhod-PE)	Avanti Polar Lipids	810150C
18:1 Phosphatidylserine (DOPS)	Avanti Polar Lipids	840035
Poly-L-Lysine	Sigma	P8920
HL5 Medium	Formedium	HLG0102
NaCl	HiMedia	PC046
Ciliobrevin-D	Sigma	250401
Adenylyl-imidodiphosphate, AMP-PNP	Sigma	10102547001
Adenosine tri-phosphate (ATP)	Sigma	34369-07-8
Guanosine triphosphate (GTP)	Sigma	G8877
Creatine-phospho Kinase	Sigma	87886
Sodium Creatine phosphate	Sigma	27920
Paclitaxel	Sigma	T1912
Polystyrene bead (500nm)	Polysciences	09836
5 micron syringe filter	Sartorius	16517E
Silicon Dioxide Microsphere (3μm)	Corpuscular	140618
Hexokinase	Sigma	H4502
Penicillin/Streptomycin	GIBCO	15140122
Phenyl methane sulfonyl fluoride (PMSF)	Sigma	T8830
DTT	Sigma	3483-12-3
Pepstatin A	MP biomedical	219536805.
Protease Inhibitor cocktail (PIC)	Sigma	11697498001
Tris	M.P. Biomedicals	819623
EGTA	Sigma	E3889
PIPES	Sigma	P1851
Pierce Trypsin protease, MS grade	Thermo fisher Scientific	90057
Iodoacetamide	Sigma	I1149
Acetic Acid, Emparta	Sigma	101830

(*Continued on next page*)

Continued		
REAGENT or RESOURCE	SOURCE	IDENTIFIER
Acetic Acid, LC-MS grade	Honeywell	49199
Water, LC-MS grade	Honeywell	39253
Acetonitrile,LC- MS grade	Fluka	34967
Formic Acid, LC -MS grade	Thermo fisher scientific	28905
Pierce C18 spin column	Thermo fisher scientific	89870
Methanol, HPLC	Sigma	34860
IPTG	Sigma	367-93-1
Lysozyme	Sigma	62971
Neomycin trisulfate salt	Sigma	N1876
Glutathione Sepharose ™ 4B	Amersham	27-4574-01
Benzamidine	Sigma	1670-14-0
Glucose	Sigma	G7021
Deposited data		
Proteomics data	This paper	PXD051777
Experimental models: Organisms/strains		
Dictyostelium discoideum	Rob Kay lab	DBS0235521
Sprague-Dawley rats	Tata Institute of Fundamental Research Mumbai	N/A
GST-KTD peptide	Kumar et al.[11]	N/A
Dictyostelium Dynein Intermediate Chain (DIC)	Sanghavi et al.[44]	N/A
Software and algorithms		
Origin-2019	OriginLab	N/A
LabView	National Instruments	N/A

RESOURCE AVAILABILITY

Lead contact

Further information and requests for resources and reagents should be directed to and will be fulfilled by the lead contact, Dr. Roop Mallik (rmallik@iitb.ac.in).

Materials availability

Materials reported in this study would be made available upon request.

Data and code availability

- Data reported in the paper would be made available upon request from the lead contact. The mass spectrometry proteomics data have been deposited to the ProteomeXchange Consortium via the PRIDE[49] partner repository with the dataset identifier PXD051777 and 10.6019/PXD051777.
- This paper does not report original code
- Any additional information required to reanalyze the data reported in this work paper is available from the lead contact upon request.

EXPERIMENTAL MODEL AND STUDY PARTICIPANT DETAILS

Animals and *Dictyostelium*

Adult 3–4 months old male Sprague-Dawley rats were used for extraction of brain. Animals were bred and housed in the Tata Institute of Fundamental Research (TIFR) animal facility in Mumbai, India. Groups of rats (3–4 animals per cage) were maintained on a 12:12 h light: dark cycle with *ad libitum* access to food and water. All experimental procedures followed the National Guidelines of the Committee for Supervision and Care of Experimental Animals (CPCSEA) and were approved by the TIFR Institutional Animal Ethics Committee. *Dictyostelium Discoideum* (AX2 axenic strain - DBS0235521) was cultured at 22°C in suspended cultures (150rpm) in HL5 medium.

METHOD DETAILS

Liposome and SSLB preparation

Phosphatidylcholine (PC; for PC liposomes) or PC and Phosphatidic acid (PA) in the molar ratio 19:1 (for PA liposomes) were dried in a nitrogen stream to form lipid films. A 0.01 M percentage of Rhodamine-phosphatidylethanolamine (Rh-PE) was added whenever needed to visualize the lipid bilayer membrane on the SSLBs. The dried lipid films were stored in a vacuum desiccator for 2 h. The lipid films were hydrated by vortexing in presence of water to form liposomes at the concentration of 1mg lipids per milliliter of distilled water, and then sonicated in a bath sonicator. These liposomes were used to make the specific types of SSLB. Washed beads, 1M NaCl solution, water, and liposomes were incubated in the ratio 5:1:69:20 (ratio of volume in ml) for 30 min with intermittent vortexing. The so-formed SSLBs were washed twice with water at room temperature for 10 min, and then used for assays within the next 10 h. All spins involving silica beads were carried out at 1000*g* and those for latex beads were carried out at 10,000*g*.

ATP-releasate preparation

Motor-enriched fractions were prepared as described in reference.[21] Briefly, *Dictyostelium* cells or Rat brain tissue were lysed using lysis buffer consisting 30% sucrose (LB-30) along with protease inhibitors. The lysate was given a low-speed (1800g) spin at 4°C for 5 min. This was followed by a high-speed spin in an MLS50 rotor (2,25,000g) on LB-30 at 4°C for 30 min. A high-speed supernatant (HSS; clarified cytosol) was collected after this spin. 360μg of goat brain tubulin was polymerized using BGT (BRB 80 + 20μM taxol +10mM GTP). Polymerized microtubules, hexokinase (15U/ml), glucose (4mM), taxol (0.2mM), AMP-PNP (1mM), and $MgCl_2$(4mM) were added to the HSS and incubated on ice for 20 min to deplete the ATP. This microtubule-cytosol mixture was centrifuged on LB-30 sucrose gradient at 1,20,000g for 20 min at 4°C. The microtubule pellet was collected and dissolved in the release buffer (LB-15 + 10mM ATP+10mM $MgCl_2$ with protease inhibitors) for 20–30 min in ice. This MT-motor mixture was spun at 1,80,000g in a TLA100 rotor for 20 min at 4°C. The supernatant was collected as ATP-releasate, aliquoted and snap snap-frozen in liquid nitrogen.

Polarity labeling of microtubules

Minus-ends of microtubules were labeled as described in.[24] Briefly, biotinylated tubulin was polymerized at 37°C for 45 min and sheared by passing through an 18G syringe needle for about 40 times. Small seeds of biotin-tubulin were mixed with unlabeled tubulin in a 1:2 ratio and polymerized at 37°C. Microtubules labeled at the minus end with biotin tubulin were then stuck to poly-lysine coated coverslips and visualized using avidin-coated magnetic beads (150nm diameter).

Motility assay and optical trapping

Plasma-cleaned coverslips were kept in a Poly-L-lysine solution (1mL Poly-L-Lysine (P8920 Sigma), 36mL ethanol, 4mL methanol) for 30 min and dried in a hot-air oven. Flow chambers are made by sticking these coverslips to a glass slide using double-stick tape (3M; 100 μm thickness). Microtubules were flowed in the chamber and incubated at room temperature for 10 min. The flow chamber was placed a microscope with a custom-built optical trap[23] and microtubules were visualized under Differential Interference Contrast (DIC). Images were acquired at 30 frames/sec. A quadrant photodiode (QPD) was used to record SSLB motion. QPD data was digitized at 2 kHz for recording SSLB motion. To calibrate the trap, thermal fluctuations of SSLBs were recorded at 40 kHz and the corner frequency was determined. Trap stiffness was adjusted between 0.05 pN/nm to 0.15 pN/nm, depending on the experiment.

5μg of ATP releasate and 3μL of SSLBs (of desired lipid composition) were incubated on ice for 5 min. For the inhibition experiments 17μM of KTD-GST, or 17μM of GST, or 20μM Ciliobrevin-D, or DMSO was added to this mix and kept on ice for an additional 5 min. This mixture, along with 1mM ATP and the ATP regenerating system (20 mM MgCl2, 40 mM creatine phosphate, and 40 U/mL creatine kinase) were flown into the flow chamber. Before adding SSLBs, coverslips were blocked with casein-taxol solution. SSLBs were trapped in the optical tweezer and placed on a single microtubule using the optical trap for 45 s. Free runs were assayed by turning the trap OFF once SSLBs bound the microtubule, whereas stalls were obtained by keeping the optical trap ON. If no motion was observed within 45 s, the SSLB was termed as non-motile.

SSLB pull-down assay

500nm latex beads were used to prepare PC or PA-SSLBs. The optical density of a given SSLB sample at 500nm wavelength (OD500) was determined. This wavelength was chosen because the Optical density of latex beads exhibits a maximum at 500nm. SSLB samples were diluted appropriately to make the OD500 equal, following which equal volumes of these "normalized" samples were used. We have characterized OD500 as a reliable parameter to normalize the number of beads (and therefore of SSLBs) across samples. For more details, see.[9,44] Such OD-normalized samples (SSLBs) have equal amount of lipids and proteins. ATP releasate containing 150μg of proteins was added to the SSLBs and incubated at room temperature for 10 min with intermittent mixing.

For the SSLB pull-down in the presence of inhibitors, 170 nM of KTD-GST peptide, or PBS, or 20μM Ciliobrevin-D, or DMSO was added to the SSLB-releasate mix and kept at room temperature for 15 min. The SSLBs were washed twice with water. The supernatant was discarded and the SSLB-bound proteins were extracted by boiling the pellet at 95°C in 0.1% SDS-containing PBS. The beads were removed by pelleting, an equal volume of the supernatant was run in an SDS gel and further processed for immunoblotting.

Microtubule pelleting assay

ATP-releasate (100μg protein content) prepared from Rat brain was incubated with 170 nM KTD-GST, or 170 nM GST peptide, or 20μM Ciliobrevin-D or DMSO for 10 min on ice. 125μg of *in vitro* polymerized Taxol-stabilized microtubule was added to this mix and incubated for 20 min on ice with intermittent mixing. The ATP releasate has ~5mM ATP. This mix was loaded onto a 25% sucrose

(with 20μM Taxol and 1mM GTP) gradient and centrifuged at 90,000g for 30 min at 4°C. The pellet was gently washed using PBS and re-suspended in 45ul 6x SDS dye. Equal volumes of these samples were loaded onto an SDS gel and the separated proteins were investigated using western blotting.

Lipid-blot assay

5 nano-moles of the specific lipids were spotted onto a nitrocellulose membrane and air-dried. The membrane was blocked using 3% BSA in TBS for 1 h at RT. The membrane was incubated overnight at 4°C with 150μg/ml of ATP releasate containing 3% BSA in TBS. The membrane was then washed thrice with 0.1% Tween containing TBS for before probing for bound motors. Primary antibody treatment was followed by secondary antibody. *Dictyostelium*-Dynein antibody (generated against Dynein Stalk Head) and Kinesin-1 antibody were used at 1:4000 for 2 h at RT in 5% skimmed milk.

Confocal imaging

PC-SSLBs and PA-SSLBs made with liposomes containing 0.01% Rhodamine-Phosphatidylethanolamine (Rhod-PE) in the lipid mixture were imaged on Zeiss 900 upright system using 63X, 1.4-NA objective.

In-gel protein digestion, peptide extraction and mass spectrometry analysis

ATP releasate

ATP releasate containing 100μg of protein was used. The releasate was mixed with 5X Laemmli buffer followed by heating at 90°C for 10 min. The sample was then loaded onto 15% polyacrylamide gel.

SSLB-bound proteins

Proteins were separated by boiling SSLBs at 95°C for 10 min in PBS containing 1% SDS. The beads were removed by pelleting. A total of 100μg of protein was used for in-gel protein digestion. The protein fraction was concentrated using centrifugal vacuum concentrator at 30°C in V-AQ mode until approximately 30μL sample volume was remaining. The protein fraction was mixed with 5X Laemmli buffer devoid of SDS, followed by heating at 90°C for 10 min prior to loading onto 15% polyacrylamide gel.

The proteins (from ATP releasate or SSLBs) were separated by SDS-PAGE until the dye front was approximately 2cm down the resolving gel. The gel was washed with dd H_2O for 5 min using an orbital shaker. The gel was fixed with gel-fixing buffer containing 10% acetic acid and 50% ethanol for 1 h at room temperature with constant shaking. The gel was then washed with gel-washing buffer containing 50% methanol and 10% acetic acid for 30 min at room temperature, followed by washing with dd H_2O for 5 min. The proteins were excise from the gel till the dye end front using razor blade, followed by cutting it into 1 mm × 1 mm small fragments. The gel fragments containing proteins were transferred to 2 mL microcentrifuge tubes. 50mM ammonium bicarbonate in 50% acetonitrile was used to wash the gel fragments for 1 h with intermittent vortexing. The gel fragments were dehydrated with 100% acetonitrile and air dried. 10mM Dithiothreitol in 50mM ammonium bicarbonate buffer was added to the dried gel pieces and incubated at 60°C for 30 min for reduction, followed by dehydration in 100% acetonitrile. The gel pieces were air dried. The gel pieces were alkylated using 20 mM iodoacetamide in 50mM ammonium bicarbonate for 30 min at room temperature in dark. The gel pieces were washed with MS grade water for 10 min to remove iodoacetamide residues. The gel pieces were dehydrated with 100% acetonitrile, followed by air drying at room temperature. To digest protein, 2μg of MS grade trypsin was added in 50mM ammonium bicarbonate to gel pieces and incubated for 1 h at 4°C to allow the gel pieces to saturate with trypsin. The sample was transferred to 37°C incubator for 18 h. The peptides were extracted using 50% acetonitrile and 0.1% formic acid, followed by 60% acetonitrile and 0.1% formic acid, 80% acetonitrile and 0.1% formic acid and 100% acetonitrile and 0.1% formic acid with 10-min vortexing, respectively. The extracted peptide solution was pooled and dried in centrifugal vacuum concentrator at 30°C in V-AQ mode. The dried peptides were resuspended in 0.1% formic acid. The tryptic peptides were desalted using pierce C18 spin column. Approximately 1 μg of tryptic peptides were analyzed using liquid chromatography coupled tandem mass spectrometry. Our analyses were performed at IIT-Bombay with a Thermo Scientific Q-Exactive plus biopharma-high resolution orbitrap connected to Thermo EASY-nLC. Peptide identities were determined using Thermo proteome discoverer 2.2.

QUANTIFICATION AND STATISTICAL ANALYSIS

ImageJ was used to quantify band intensities of western blots and the backgrounds. Origin-2019 was used to prepare the graphs. Custom developed software in LabView (National Instruments) was used to visualize optical trap-generated QPD data. Wherever data was known to be normally distributed, a two-tailed Student's *t-test* was used to test the significance of the results (95% confidence interval).

Cell Reports, Volume *43*

Supplemental information

Phosphatidic acid-dependent recruitment of microtubule motors to spherical supported lipid bilayers for *in vitro* motility assays

Pankaj Kumar, Dwiteeya Chaudhury, Paulomi Sanghavi, Apurwa Meghna, and Roop Mallik

SUPPLEMENTARY INFORMATION

Supplementary Figures 1 and 2
Supplementary Table 1

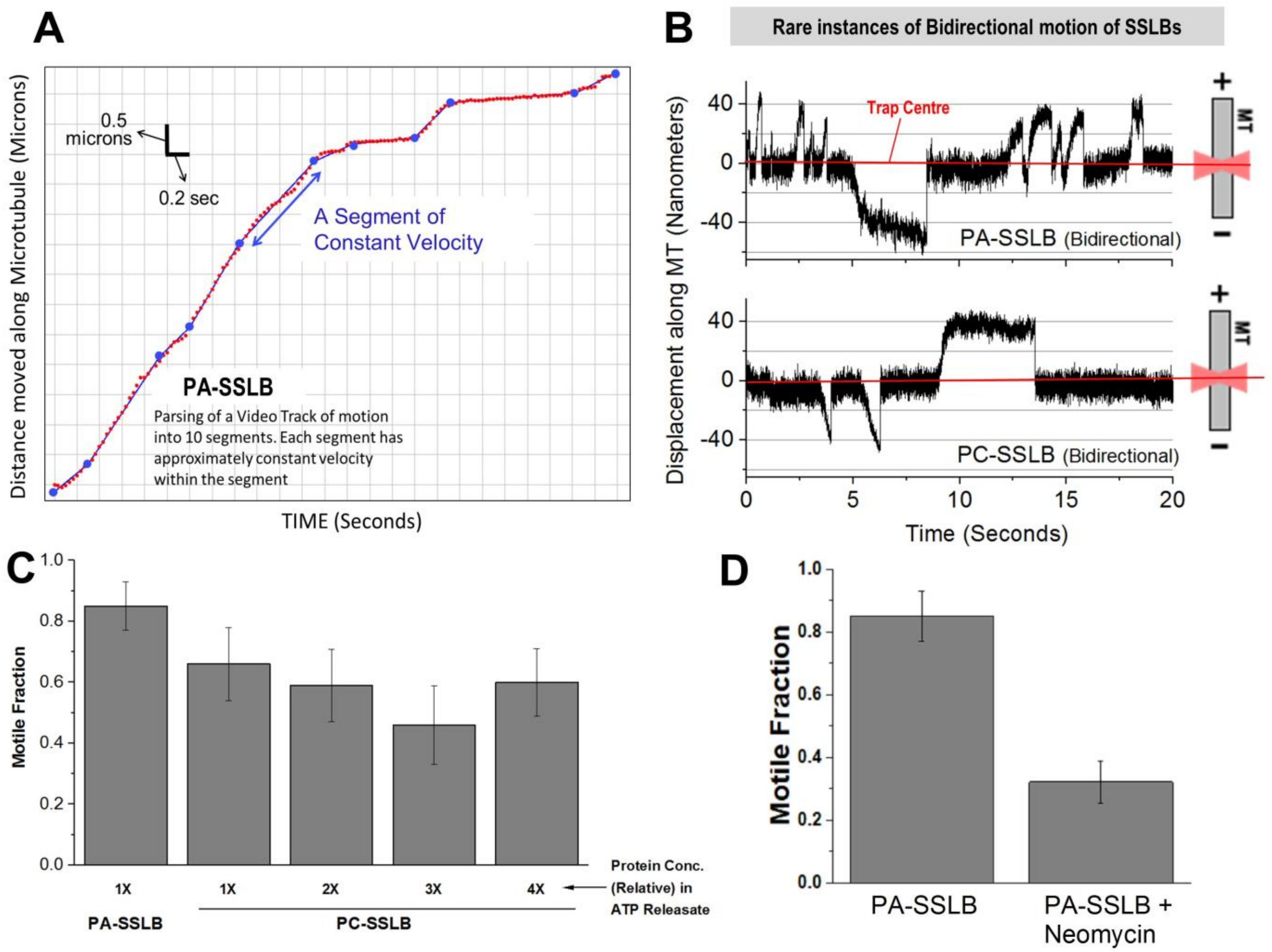

Supplementary Figure 1

Motion of SSLBs under different conditions

A. Distance versus time track (red dots) of a PA-SSLB moving freely along a MT (trap switched OFF). A Bayesian algorithm is applied to parse this track into 10 segments, each having an approximately constant velocity within the segment. The parsed segments are shown as large blue dots connected by a blue line. One such segment having a velocity (= slope) of 2 microns/sec is shown. The velocities obtained from such parsing are used in Figs 1B and 2B.

B. Bidirectional displacements against an optical trap for a PA-SSLB (top) and a PC-SSLB (bottom) incubated with *Dictyostelium* ATP releasate. These are the rare incidents where a given SSLB showed excursions in both directions against the trap. Red line represents the trap center.

C. Motile fraction of PA-SSLBs in presence of 1X (= 5µg total protein) amount of *Dictyostelium* ATP releasate. PC-SSLBs are incubated with increasing amounts of ATP releasate (1X to 4X; 5µg to 20µg protein). Error = $\sqrt{(P*(1-P)/N)}$. *P* is the motile fraction and *N* the number of SSLBs tested.

D. Motile fraction of PA-SSLBs in presence and absence of Neomycin (1mM). Error = $\sqrt{(P*(1-P)/N)}$. *P* is the motile fraction and *N* the number of SSLBs tested.

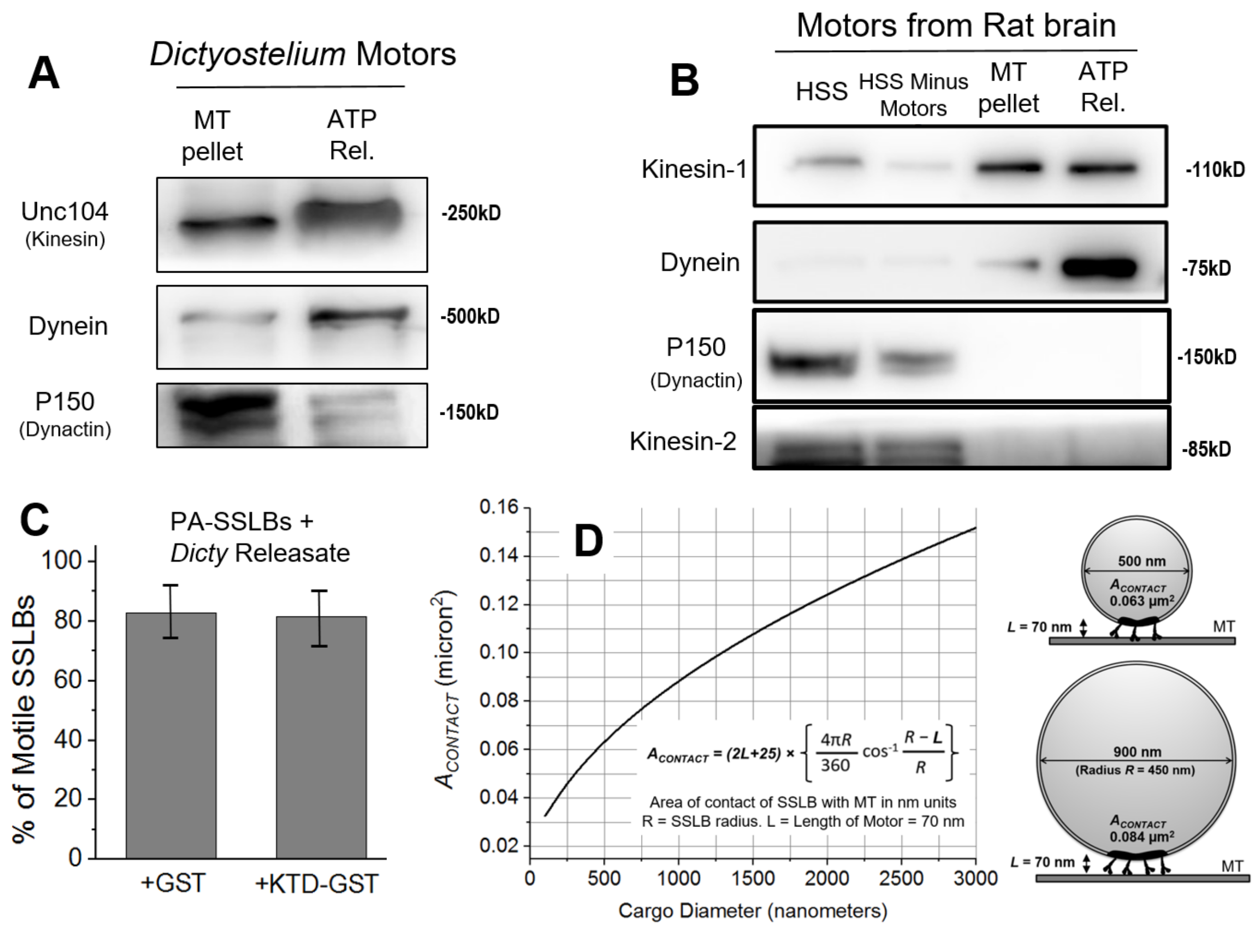

Supplementary Figure 2

Dynein, Dynactin, Kinesins probed in biochemical fractions, effect of KTD on *Dictyostelium* Motors, and Contact area of Motors on SSLBs.

A. Western blots probed for Dynein, Unc104, and Dynactin (P150) in the MT pellet and ATP releasate fractions from *Dictyostelium*. **MT pellet** is the pellet containing MTs after the ATP-release step has been done (see next). **ATP Rel.** is the supernatant after MTs have been pelleted down in the presence of 10mM ATP. ATP releasate is the motor-enriched fraction that is added to SSLBs in the motility assay. The MT pellet and ATP releasate lanes contain 75µg total protein per lane.

B. Western blots probed for Kinesin-1, Dynein, Dynactin (P150), and Kinesin-2 in different fractions obtained during the preparation of ATP releasate from rat brain. **HSS** High-speed supernatant (= clarified cytosol) after pelleting the cell membranes. *In-vitro* polymerized MTs are added to this HSS for binding motors and MAPs. **HSS Minus Motors** is the supernatant obtained after MT-bound motors and MAPs are pelleted down from the HSS. Equal volumes of HSS and HSS-Motors were loaded. **MT pellet** is the pellet containing MTs after the ATP-release step has been done (see next). **ATP Rel.** is the supernatant after MTs have been pelleted down in the presence of 10mM ATP. The MT pellet and ATP releasate lanes contain 15µg total protein per lane.

C. Motile fraction of PA-SSLBs prepared from *Dictyostelium* releasate in presence of control (GST) and Kinesin Tail Domain (KTD-GST). Error = $\sqrt{(P*(1-P)/N)}$. *P* is the motile fraction and *N* the number of SSLBs tested.

D. Graph represents change in $A_{CONTACT}$ with diameter of a spherical cargo (e.g. SSLB). Cartoon insets are schematics representing $A_{CONTACT}$, which is the region on a spherical cargo from where a motor of given size (L =70 nm) can access the underlying MT. The expression for $A_{CONTACT}$ is shown. See *Sanghavi et al Current Biology 2018* for more details. The two cargoes of different size and their attached motors are drawn to relative scale. Motors outside $A_{CONTACT}$ are not shown for the sake of clarity.

Supplementary Table 1

Selected Proteins detected by LC-MS/MS		Number of Unique Peptides detected		
**Accession Number**	**Description**	**ATP Releasate**	**PA-SSLBs (Sample 1)**	**PA-SSLBs (Sample 2)**
P05095	Alpha-actinin A	32	31	31
P54703	Cytoplasmic dynein 1 intermediate chain	3	8	11
Q54CI8	Cytoplasmic dynein 1 light intermediate chain 1	6	7	9
Q54VD6	Dynactin 150 kDa subunit	7	30	29
Q54N50	Dynactin subunit 2	7	13	16
Q55F94	Dynamin-like protein A	16	26	27
Q54MH8	Dynamin-like protein B	8	13	16
Q54VS0	Dynactin subunit 4	3	3	4
P34036	Dynein heavy chain, cytoplasmic	77	153	174
Q9NGQ2	Kinesin-related protein 1	57	79	97
Q6S001	Kinesin-related protein 11	6	8	12
Q54UC9	Kinesin-related protein 3	28	38	34
Q8T135	Kinesin-related protein 5	11	15	17
Q6S003	Kinesin-related protein 8	9	19	25
P32255	Tubulin alpha chain	7	15	13
P32256	Tubulin beta chain	11	16	18

Supplementary Table 1.

LC-MS/MS proteomics of ATP releasate from *Dictyostelium* and PA-SSLBs incubated with ATP releasate.

Selected proteins that were detected using mass spectrometry (LC-MS/MS) in ATP releasate from *Dictyostelium* and on PA-SSLBs incubated with ATP releasate from *Dictyostelium.* PA-SSLBs were prepared, tested and analysed in duplicate (Sample 1 and Sample 2). A complete list of detected proteins is available from the PRIDE database with the dataset identifier PXD051777.
